# Metabolic signatures of greater body size and their associations with risk of colorectal and endometrial cancers in the European Prospective Investigation into Cancer and Nutrition

**DOI:** 10.1186/s12916-021-01970-1

**Published:** 2021-04-30

**Authors:** Nathalie Kliemann, Vivian Viallon, Neil Murphy, Rebecca J. Beeken, Joseph A. Rothwell, Sabina Rinaldi, Nada Assi, Eline H. van Roekel, Julie A. Schmidt, Kristin Benjaminsen Borch, Claudia Agnoli, Ann H. Rosendahl, Hanna Sartor, José María Huerta, Anne Tjønneland, Jytte Halkjær, Bas Bueno-de-Mesquita, Audrey Gicquiau, David Achaintre, Krasimira Aleksandrova, Matthias B. Schulze, Alicia K. Heath, Konstantinos K. Tsilidis, Giovanna Masala, Salvatore Panico, Rudolf Kaaks, Renée T. Fortner, Bethany Van Guelpen, Laure Dossus, Augustin Scalbert, Hector C. Keun, Ruth C. Travis, Mazda Jenab, Mattias Johansson, Pietro Ferrari, Marc J. Gunter

**Affiliations:** 1 International Agency for Research on Cancer, World Health Organization, Lyon, France; 2 Leeds Institute of Health Sciences, University of Leeds, Leeds, UK; 3 Department of Behavioural Science and Health, University College London, London, UK; 4 Health Across Generations team, Centre for Research in Epidemiology and Population Health (CESP), INSERM U1018, Villejuif, France; 5 Gustave Roussy, F-94805 Villejuif, France; 6 Department of Epidemiology, GROW School for Oncology and Developmental Biology, Maastricht University, Maastricht, The Netherlands; 7 Cancer Epidemiology Unit, Nuffield Department of Population Health, University of Oxford, Oxford, UK; 8 Department of Community Medicine, Faculty of Health Sciences, UiT, The Arctic University of Norway, Tromsø, Norway; 9 Epidemiology and Prevention Unit. Fondazione IRCCS Istituto Nazionale dei Tumori, Milan, Italy; 10 Clinical Sciences Lund, Oncology, Lund University and Skåne University Hospital, Lund, Sweden; 11 Diagnostic Radiology, Lund University, Lund, Sweden; 12 CIBER Epidemiología y Salud Pública (CIBERESP), Madrid, Spain; 13 Department of Epidemiology, Murcia Regional Health Council, IMIB-Arrixaca, Murcia, Spain; 14 Danish Cancer Society Research Center, Copenhagen, Denmark; 15 Department for Determinants of Chronic Diseases, National Institute for Public Health and the Environment, Bilthoven, The Netherlands; 16 Nutrition, Immunity and Metabolism Senior Scientist Group, Department of Nutrition and Gerontology, German Institute of Human Nutrition Potsdam-Rehbruecke (DIfE), Nuthetal, Germany; 17 Institute of Nutritional Science, University of Potsdam, Potsdam, Germany; 18 Department of Molecular Epidemiology, German Institute of Human Nutrition Potsdam-Rehbruecke, Nuthetal, Germany; 19 Department of Epidemiology and Biostatistics, School of Public Health, Imperial College London, London, UK; 20 Department of Hygiene and Epidemiology, University of Ioannina School of Medicine, Ioannina, Greece; 21 Cancer Risk Factors and Life-Style Epidemiology Unit, Institute for Cancer Research, Prevention and Clinical Network – ISPRO, Florence, Italy; 22 Dipartimento di Medicin Clinica e Chirurgia, Frederico II Univeristy, Naples, Italy; 23 Division of Cancer Epidemiology, German Cancer Research Center (DKFZ), 69120 Heidelberg, Germany; 24 Department of Radiation Sciences, Oncology, Wallenberg Centre for Molecular Medicine, Umeå University, Umeå, Sweden; 25 Cancer Metabolism and Systems Toxicology Group, Division of Cancer, Department of Surgery and Cancer, Imperial College, London, UK

**Keywords:** Metabolomics, Obesity, Weight loss, Cancer

## Abstract

**Background:**

The mechanisms underlying the obesity-cancer relationship are incompletely understood. This study aimed to characterise metabolic signatures of greater body size and to investigate their association with two obesity-related malignancies, endometrial and colorectal cancers, and with weight loss within the context of an intervention study.

**Methods:**

Targeted mass spectrometry metabolomics data from 4326 participants enrolled in the European Prospective Investigation into Cancer and Nutrition (EPIC) cohort and 17 individuals from a single-arm pilot weight loss intervention (Intercept) were used in this analysis. Metabolic signatures of body size were first determined in discovery (*N* = 3029) and replication (*N* = 1297) sets among EPIC participants by testing the associations between 129 metabolites and body mass index (BMI), waist circumference (WC), and waist-to-hip ratio (WHR) using linear regression models followed by partial least squares analyses. Conditional logistic regression models assessed the associations between the metabolic signatures with endometrial (*N* = 635 cases and 648 controls) and colorectal (*N* = 423 cases and 423 controls) cancer risk using nested case-control studies in EPIC. Pearson correlation between changes in the metabolic signatures and weight loss was tested among Intercept participants.

**Results:**

After adjustment for multiple comparisons, greater BMI, WC, and WHR were associated with higher levels of valine, isoleucine, glutamate, PC aa C38:3, and PC aa C38:4 and with lower levels of asparagine, glutamine, glycine, serine, lysoPC C17:0, lysoPC C18:1, lysoPC C18:2, PC aa C42:0, PC ae C34:3, PC ae C40:5, and PC ae C42:5. The metabolic signature of BMI (OR_1-sd_ 1.50, 95% CI 1.30–1.74), WC (OR_1-sd_ 1.46, 95% CI 1.27–1.69), and WHR (OR_1-sd_ 1.54, 95% CI 1.33–1.79) were each associated with endometrial cancer risk. Risk of colorectal cancer was positively associated with the metabolic signature of WHR (OR_1-sd_: 1.26, 95% CI 1.07–1.49). In the Intercept study, a positive correlation was observed between weight loss and changes in the metabolic signatures of BMI (*r* = 0.5, 95% CI 0.06–0.94, *p* = 0.03), WC (*r* = 0.5, 95% CI 0.05–0.94, *p* = 0.03), and WHR (*r* = 0.6, 95% CI 0.32–0.87, *p* = 0.01).

**Conclusions:**

Obesity is associated with a distinct metabolic signature comprising changes in levels of specific amino acids and lipids which is positively associated with both colorectal and endometrial cancer and is potentially reversible following weight loss.

**Supplementary Information:**

The online version contains supplementary material available at 10.1186/s12916-021-01970-1.

## Background

Obesity is an important risk factor for at least 13 different types of cancer [1]. It is estimated that 3.9% (~ 550,000 cases in 2012) of the total worldwide cancer burden is related to obesity, while 5.7% (~ 800,000 cases in 2012) is attributed to both obesity and type 2 diabetes [1, 2]. Experimental and molecular epidemiologic studies indicate important roles for dysregulated sex hormone metabolism, adipose tissue-derived inflammation, and alterations in insulin signalling in mediating the adiposity and cancer associations [3, 4]. However, it is likely that other, as of yet unidentified, biological pathways may also underlie these relationships. Further, it is not clear whether weight loss promotes changes in the metabolic pathways linking obesity and cancer development, and ultimately whether it lowers cancer risk.

Metabolomics is an established technology for the identification of metabolic changes and biomarkers for understanding pathophysiological processes, through simultaneous measurement of multiple metabolites in human biofluids or tissues [5, 6]. Metabolomic profiling has the potential to identify specific metabolic phenotypes that are associated with cancer and to provide insights into the mechanistic pathways involved in cancer development [7–9].

To date, a limited number of epidemiological studies have identified metabolic and biochemical pathways that are significantly altered in obesity [10–12]. In a previous analysis in the European Prospective Investigation into Cancer and Nutrition (EPIC) cohort, a metabolic signature of high body mass index (BMI) was positively associated with hepatocellular carcinoma risk (HCC) and was found to mediate much of the association between measured BMI and HCC [13]. However, to date, no study has linked metabolic signatures reflecting other anthropometric measures of obesity, such as waist circumference (WC) and waist-to-hip ratio (WHR), to obesity-related cancer development. Similarly, very few studies have investigated the biochemical pathways altered during weight loss [14, 15]. The Intercept study, a pilot intervention promoting weight loss through meal replacement diet among individuals with obesity, was one of the first studies to demonstrate potential cancer-relevant changes in colorectal tissue following substantial weight loss [16].

In this analysis, we identified metabolic signatures associated with greater body sizes as determined by BMI, WC, and WHR, and then investigated their association with risk of colorectal and endometrial cancers—two malignancies strongly linked to obesity and metabolic dysfunction [1]—in EPIC. We also explored the extent to which weight loss modified these metabolic signatures within a single-arm weight loss intervention study.

## Methods

### European Prospective Investigation into Cancer and Nutrition

EPIC is a multicentre cohort of 521,330 participants who were recruited between 1991 and 2000, predominantly from the general populations of 10 European countries (Denmark, France, Germany, Greece, Italy, the Netherlands, Norway, Spain, Sweden, and the UK) [17, 18]. The current study used data from all EPIC countries apart from Greece.

Anthropometric characteristics were measured by trained observers using standardised methods [18]. Body weight was measured in all centres by electronic digital scales, with participants wearing only light underwear and after voiding the bladder. Height was measured to the nearest 0.1 cm using a flexible anthropometer [19]. The exceptions were Oxford, France, and Norway where these measures were self-reported; however, they were shown to be valid for identifying associations in epidemiological studies [20, 21]. Assessed weight and height (measured and self-reported) were used to calculate body mass index (BMI) defined as weight in kilogrammes divided by height in metres squared (kg/m^2^). Waist circumference (WC) was measured either at the narrowest torso circumference or at the midpoint between the lower ribs and iliac crest. At baseline, questionnaires were used to collect information on demographics, behavioural factors including dietary intakes, and medical information. Physical activity levels were estimated using a questionnaire focused on past-year physical activity in occupational, leisure, and household domains and classified according to the validated Cambridge physical activity index [22]. Validated country/centre-specific dietary questionnaires were used to obtain information on dietary intake including total energy, dietary fibre, fish and shellfish, meat, and processed meat intake.

Incident cancer cases were identified using cancer registries in Norway, Sweden, UK, Spain, Italy, the Netherlands, and Denmark. For France and Germany, incident cancer cases were identified during follow-up from a combination of sources including cancer and pathology centres, health insurance records, and active follow-up of study subjects. All countries followed a detailed protocol for the collection and standardisation of clinical and pathological data on each cancer site [23–26]. Cancer cases were defined using the tenth revision of the International Classification of Diseases (ICD-10) (ICD-10) and the second revision of the International Classification of Diseases for Oncology (ICDO-2). In the current analysis, we focused on two malignancies strongly linked to obesity and metabolic dysfunction [1], colorectal (C180-209) and endometrium (C540-549), more specifically on type I (endometrioid type) endometrial tumours, and on colon cancer.

#### Study participants

For the derivation of the metabolic signature, we used existing data from 4326 participants who had been selected as matched control (non-cancer) participants in four separate case–control studies nested within EPIC (breast, kidney, liver and prostate cancer studies). These control participants were selected as they had both metabolomics and anthropometric data available. These data were randomly split into a discovery set (*N* = 3029) and a replication set (*N* = 1297), for the validation of the metabolic signatures. The derived metabolic signatures associated with greater body size were then investigated in relation to colorectal and endometrial cancers using data from two case-control studies nested within EPIC comprising 423 colorectal cancer cases and 423 matched controls and 635 endometrial cancer cases and 648 matched controls. For the colorectal case-control study, participants were matched on study recruitment centre, sex, age at blood collection, time of blood collection, and fasting status. Similar matching criteria were applied to the endometrial case-control study, but also included menopausal status, and for premenopausal women, phase of menstrual cycle. All participants included in the current study self-reported being free of diabetes and not using hormone replacement therapy at baseline (women only).

### Intercept weight loss pilot intervention

The Intercept study was a single-arm pilot study testing the effect of weight loss among obese individuals on biomarkers of colorectal cancer risk measured in serum and colorectal tissue biopsies. The study was registered on the ISRCTN registry as ISRCTN35702367. Twenty-six participants (21–57 years old) with obesity (BMI ≥ 30 kg/m^2^) were recruited via advertisements placed around University College London between July 2013 and July 2014. Briefly, participants followed an 8-week liquid weight-loss diet programme (810 cal per day) based on formula diet products (Cambridge Weight Plan™, Northants., UK). A detailed description of the study and primary results has been published elsewhere [16]. The programme was carried out by trained researchers, who also offered support and advice on behaviour change techniques on a weekly basis. At the end of the 8 weeks, 4 weeks of additional support were provided to help participants with meal reintroduction and weight loss maintenance. Pre- and post-intervention measures of weight, height, and waist circumference were collected, and two 20-ml fasting blood (serum) samples were taken by a research nurse at each time point.

#### Study participants

Data from 17 participants from the Intercept pilot intervention with metabolomics data measured in serum samples collected pre- and post-intervention were included to assess the association between the derived body size-related metabolic signatures and weight loss.

### Laboratory analysis

Plasma (EPIC) and serum (Intercept) metabolites from all study populations were assayed using the targeted AbsoluteIDQ p180 kit (BIOCRATES Life Sciences AG, Innsbruck, Austria) on the liquid chromatography mass spectrometry (LC/MS) platform at IARC, Lyon, France. The exception were the plasma metabolites from the endometrial case-control study, which were assayed at Imperial College London, UK, using the same methodology. Amino acids and biogenic amines were separated by liquid chromatography before injection into the mass spectrometer, while flow injection analysis was used for glycerophospholipids, hexoses, acylcarnitines, and sphingolipids. Metabolites with inter-batch or intra-batch coefficients of variation (CVs) larger than 20% for analytical replicates and with more than 20% missing data were excluded. For the included metabolites, measurements below the limit of detection (LOD) or quantification (LOQ) were set to half the batch-specific LOD or LOQ, respectively. When all of the samples within the same batch had measurements below the LOD and LOQ, half of the lowest measured concentration detected for that metabolite across all other batches was imputed. All metabolites above the highest calibration standard were given the highest value obtained in the sample. For the metabolites with up to 20% missing data, the median value was imputed. In the EPIC discovery and replication sets, 129 metabolites were included. For the colorectal, endometrial and Intercept data sets 129, 124, and 128 metabolites were included, respectively (Additional file 1: Table S1).

C-peptide was assayed in serum samples from a sample of EPIC participants by enzyme-linked immunosorbent assay by Mercodia (Sylveniusgatan, Sweden) as previously described [27]. The mean intra-batch and inter-batch coefficients of variation were 6.69% and 5.75%, respectively, for C-peptide concentration of 5 ng/ml [27].

### Statistical analysis

Descriptive analyses were performed for sociodemographic, behavioural and blood sampling-related variables for each study population (discovery and replication sets, colorectal and endometrial nested case-control studies and Intercept pilot intervention). Within each study population, metabolite levels were log-transformed (natural logarithm) and Z-standardised.

The Principal Component Partial R-square (PC-PR2) method [28] was performed in each study population to estimate the contribution to total variability in metabolite levels attributed to anthropometric variables and other factors such as subjects’ characteristics and technical aspects of the samples. In the EPIC discovery set, lifestyle (physical activity, smoking status and dietary intake), fasting status, sex, age at blood collection, and batch/study variables all together explained 32% of the total variability in metabolite levels (Additional file 1: Figure S1, Figure S2 and Figure S3). The main contributions of variability were batch/sub-study (8%) and country (13%). Regarding the anthropometrics, BMI, WC, and WHR explained 1.34%, 1.36%, and 1.10% of the total variability, respectively. Similar variability was observed in the replication set and in the colorectal and endometrial case-control studies. In the Intercept study, the variability of the changes in metabolites over 8 weeks were mainly explained by percentage of weight loss (32%), while baseline BMI, WC, and WHR explained 15%, 17%, and 13%, respectively (Additional file 1: Figure S4).

In all study populations, the metabolite levels (Z-standardised and log-transformed) were transformed into residuals of linear models with country and sex as independent variables (when possible) and random intercepts for analytical batches (nested within studies, when relevant).

#### Metabolic signatures of greater body sizes

In the discovery and replication sets, the residuals were used as dependent variables in linear regression models testing confounder-adjusted associations with log-transformed BMI, WC, and WHR. Models were adjusted for a set of a priori-defined covariates that included age at blood collection (continuous), fasting status at blood collection (< 3 h/3–6 h/> 6 h/unknown), education, smoking status at recruitment (current/former/never/unknown), physical activity index (inactive/moderately inactive/moderately active/active/unknown), height, and daily intake of energy, red and processed meat products, fish and shellfish, fibre, and alcohol (all continuous). In the discovery analysis, false discovery rate (FDR) adjustment of *p* values was applied using the Benjamini–Hochberg method (*q* values < 0.05 were considered statistically significant), as it has greater power to detect real differences when compared to the Bonferroni approach and reduces the risk that potentially relevant metabolites might be missed in the discovery phase. Then, in the replication phase, in which we eliminate the non-relevant metabolites and we focus on a smaller set of metabolites (only those that were significant in the discovery analysis), we used a more conservative approach, that is, Bonferroni correction (in order to only select the metabolites that are most highly statistically significantly associated with the outcome). Separate models were run for each exposure, i.e. BMI, WC, and WHC. In the discovery set, the PC-PR2 method was performed again using residuals of each metabolite in which its association with body size was validated. Also in the discovery set, residuals of each metabolite in which its association with body size was validated were included as multivariate predictors of greater body size in the PLS regression, a multivariate method that achieves dimensionality reduction [29]. The metabolic signatures were predicted in the replication set and correlated to BMI, WC, and WHR in the entire sample as well as separately by sex (Pearson correlation was applied).

#### Metabolic signatures of greater body sizes and cancer risk

The metabolic signatures of greater body size were predicted in all participants from each nested case-control study using the residuals for each metabolite. Conditional logistic regression models were applied to assess the associations between the metabolic signatures of greater body size and risk for colorectal and endometrial cancers. The multivariable model included adjustment for age at blood collection, fasting status at blood collection, education, smoking status at recruitment, physical activity index, height, and daily intake of energy, red and processed meat products, fish and shellfish, fibre, and alcohol. For colorectal cancer, the models were further adjusted for fibre and calcium intake, while for endometrial cancer the models were further adjusted for menopause status (premenopausal, perimenopausal, and postmenopausal), age at first menstrual period, age at first full-term pregnancy, hormone therapy and oral contraceptive use. In an attempt to investigate whether the metabolic signatures were able to predict cancer risk beyond individuals’ body size, the multivariate models were further adjusted for BMI and residuals of the linear regression of WC on BMI and the residuals of the linear regression of WHR on BMI and WC. We have also further adjusted the models for C-peptide level, a valid marker of insulin secretion, as a possible confounder between the associations of metabolic signatures and cancer risk. Further adjustment for cancer grade and stage was also conducted. Additionally, we investigated the association between the log-transformed anthropometric variables and cancer risk. Correlations between the metabolic signatures and BMI, WC, and WHR among colorectal and endometrial controls were also tested. Finally, sensitivity analyses were performed by excluding cases of cancer diagnosed during the first two years of each participant’s follow-up to minimise/limit reverse causality bias.

#### Metabolic signatures of greater body size and weight loss

In the intercept pilot intervention, paired *t* tests were employed to determine which metabolites were significantly altered during the intervention. Changes in metabolite levels post-versus pre-intervention were calculated and were further transformed into residuals of linear models with sex as independent variable. The metabolic signatures of greater body size were predicted in all participants for the Intercept study using the residuals for metabolite changes and described by tertiles of weight loss. This approach allowed us to assess changes in the metabolic signatures as it provides results similar to calculating the difference between the metabolic signature at follow-up and at baseline. Pearson correlations between changes in metabolic signatures of body sizes and percentage of weight loss were assessed. Additionally, linear regression models tested associations between residuals for metabolite changes and weight loss adjusted for age and residuals for anthropometric variables. Statistical tests were two-sided and *P* values *<* 0.05 were considered statistically significant. All analyses were performed in Stata 15 and R 3.6.3 statistical software. A flow-diagram with the main methodological steps undertaken in the current study is presented in supplementary material (Additional file 1: Figure S5).

## Results

Sociodemographic and behavioural characteristics of participants in each study population are presented in Table 1. The majority of the participants in the EPIC discovery and replication sets were men (~ 70%), while in the colorectal case-control (~ 60%), endometrial case-control (100%), and Intercept (~ 65%) studies, most participants were women. In the EPIC discovery set, around 36% of participants were normal weight and 64% were overweight or obese and similar characteristics were observed in the other EPIC sets. Participants were followed-up by a mean of 10.9 (SD 5.2) and 12.1 (SD 4.9) years in the colorectal and endometrial case-control studies, respectively. Cases and controls showed similar characteristics. In the Intercept pilot intervention, all participants were obese and lost at least 10% of their initial body weight (mean = 13.4 kg).

**Table 1:** Sociodemographic, lifestyle, dietary and blood-sampling related characteristics of participants in the study populations

	European Prospective Investigation into Cancer and Nutrition						Intercept*
	Discovery	Replication	Colorectal cancer		Endometrial cancer		
			Cases	Controls	Cases	Control	
	(*n* = 3029)	(*n* = 1297)	(*n* = 423)	(*n* = 423)	(*n* = 635)	(*n* = 648)	(*n* = 17)
**Age, mean (SD)**	55.5 (8.1)	55.8 (8.2)	55.7 (7.8)	55.8 (7.9)	53.96 (7.9)	53.99 (7.9)	37.5 (9.7)
**Sex,** ***n*** **(%)**							
Women	964 (31.8)	431 (33.2)	257 (60.8)	253 (59.9)	635 (100)	648 (100)	11 (64.7)
**Educational level,** ***n*** **(%)**							
University or higher	674 (22.2)	296 (22.8)	62 (14.6)	65 (15.3)	116 (18.2)	92 (14.2)	13 (76.4)
**Body mass index (kg/m**^**2**^**), mean (SD)**	26.45 (3.7)	26.47 (3.7)	27.1 (4.5)	26.51 (3.6)	28 (5.4)	25.85 (4.2)	34.2 (3.8)
**Categories of BMI,** ***n*** **(%)**							
Normal weight	1101 (36.3)	467 (36.0)	142 (33.6)	141 (33.3)	210 (33.1)	315 (48.6)	–
Overweight	1456 (48.1)	619 (47.7)	182 (43.0)	216 (51.0)	226 (35.6)	239 (36.8)	1 (5.8)
Obese	472 (15.6)	211 (16.3)	99 (23.4)	66 (15.6)	199 (31.3)	94 (14.5)	16 (94.2)
**Waist circumference (cm), mean (SD)**	90.7 (12.0)	90.4 (12.1)	89.6 (13.3)	87.8 (11.5)	85.7 (12.1)	81.5 (10.5)	102.8 (8.0)
**Waist-hip ratio (cm), mean (SD)**	0.89 (0.09)	0.89 (0.09)	0.87 (0.09)	0.86 (0.1)	0.81 (0.06)	0.80 (0.06)	0.86 (0.7)
**Height (cm), mean (SD)**	169.0 (8.8)	168.9 (8.9)	164.3 (9.1)	163.8 (9.4)	160.2 (6.6)	160.2 (6.8)	170.6 (7.9)
**Physical activity,** ***n*** **(%)**							
Inactive	679 (22.4)	296 (22.8)	133 (31.4)	122 (28.8)	95 (14.9)	86 (13.3)	NA
Moderately inactive	1026 (33.9)	463 (35.7)	183 (43.2)	175 (41.4)	189 (29.7)	175 (27.0)	NA
Moderately active	670 (22.1)	278 (21.4)	63 (14.9)	71 (16.8)	18 (2.8)	13 (2.0)	NA
Active	597 (19.7)	237 (18.3)	44 (10.4)	54 (12.7)	115 (18.1)	126 (19.4)	NA
**Smoking status,** ***n*** **(%)**							
Never smoker	1224 (40.4)	518 (39.9)	198 (46.8)	222 (52.5)	82 (12.9)	111 (17.1)	NA
Current smoker	703 (23.2)	299 (23.0)	90 (21.3)	77 (18.2)	417 (65.6)	396 (61.1)	NA
**Meat intake (g/d), mean (SD)**	86.5 (55.2)	88.9 (59.5)	76.7 (81.7)	77.6 (44.4)	634.7 (378.8)	646.2 (376.4)	NA
**Fish intake (g/d), mean (SD)**	37.1 (37.2)	35.3 (33.7)	36.3 (32.4)	37.9 (36.5)	523.9 (316.5)	509.0 (312.9)	NA
**Fibre intake (g/d), mean (SD)**	23.7 (8.1)	23.3 (7.8)	22.9 (7.6)	23.3 (8.0)			NA
**Alcohol intake (g/d), mean (SD)**	3.1 (1.3)	3.1 (1.27)	2.9 (1.4)	2.7 (1.3)	3.1 (1.2)	3.1 (1.2)	NA
**Energy intake (g/d), mean (SD)**	2253 (654.7)	2255 (643.0)	2221 (829.0)	2187 (627.9)	661 (383.9)	667 (380.1)	NA
**Fasting status,** ***n*** **(%)**							
Non-fasting	1351 (44.6)	601 (46.3)	23 (5.4)	23 (5.4)	99 (15.6)	105 (16.2)	17 (100)
**Sub-study,** ***n*** **(%)**							
Breast cancer controls	752 (24.8)	332 (25.6)	n/a	n/a	n/a	n/a	n/a
Kidney cancer controls	346 (11.4)	134 (10.3)	n/a	n/a	n/a	n/a	n/a
Hepatobiliary cancer controls	151 (4.9)	65 (5.0)	n/a	n/a	n/a	n/a	n/a
Prostate cancer controls	1780 (58.7)	766 (59.0)	n/a	n/a	n/a	n/a	n/a

*Intercept pilot weight loss intervention. NA, not available; n/a, not applicable

### Metabolic signatures of greater body sizes

**Fig. 1:**
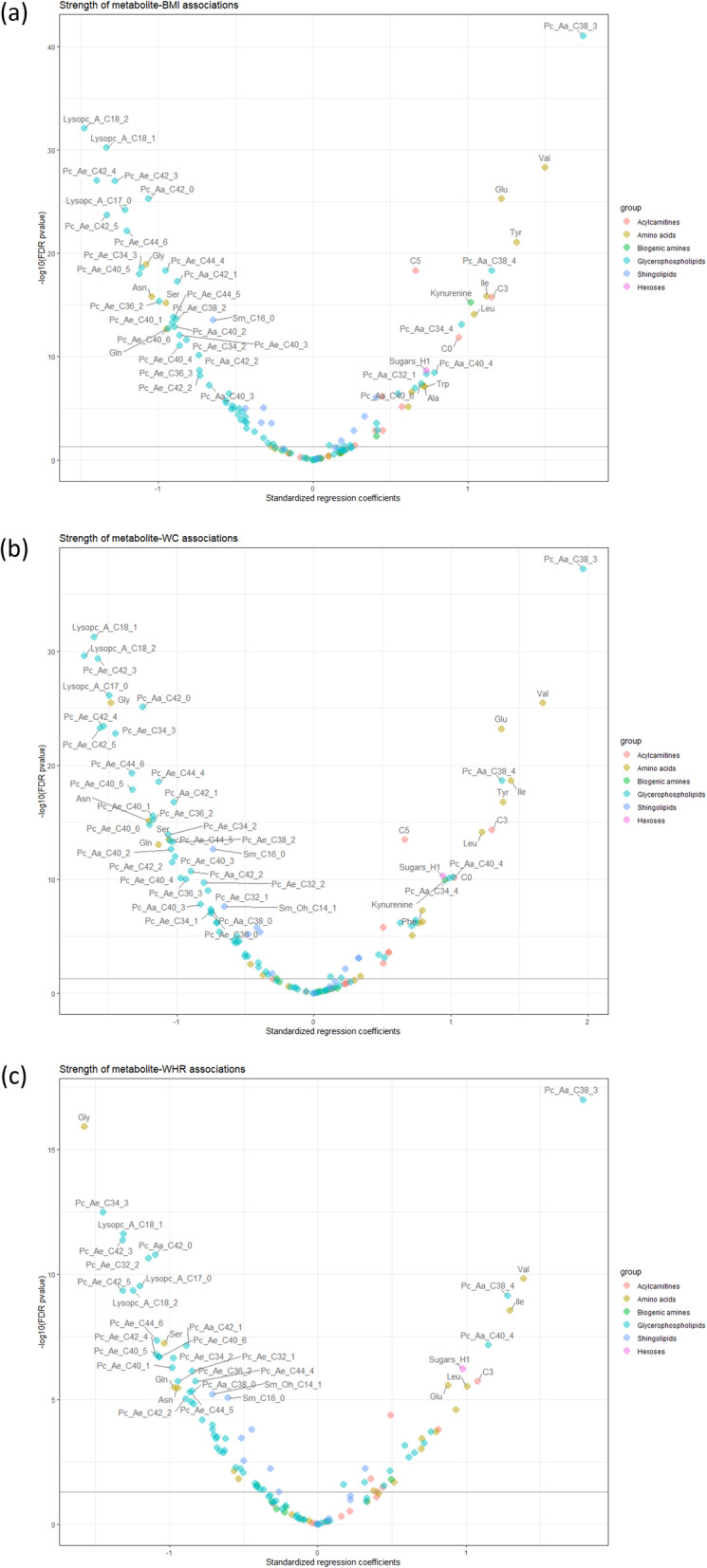
Smile plot with associations between metabolites with BMI, WC and WHR in the discovery set. **a** BMI, **b** WC, and **c** WHR. Smile plot with FDR (false discovery rate method) *q* values. Analysis using residuals from Z and Log transformed metabolites with fixed effect for country and sex and random effect for batches nested within studies. Models were adjusted for age at blood collection, fasting status at blood collection, smoking status at recruitment, Cambridge physical activity index, height, and daily intake of energy, red and processed meat, fish and shellfish, fibre, and alcohol. The metabolites above the horizontal line showed a significant association with the anthropometric measure (*p* < 0.05)

In the discovery set, BMI, WC, and WHR were statistically significantly (after FDR-adjustment; degrees of freedom = 2949) associated with levels of 89, 94, and 75 metabolites, respectively (Fig. 1). In the replication phase, after Bonferroni correction, 47% (*N* = 42), 42% (*N* = 40), and 21% (*N* = 16) of these metabolites were associated with BMI, WC, and WHR, respectively (Additional file 1: Table S2). As shown in Table 2, a total of 16 metabolites were associated with all three measures, and 23 were associated with both BMI and WC. Considering the metabolites with the strongest association with the three anthropometric measures, higher BMI, WC, and WHR were associated with higher levels of valine, phosphatidylcholine diacyl (PC aa) C38:3, and lower levels of lysophosphatidylcholine acyl (LysoPC) C18:2. PC-PR2 was performed using residuals of each metabolite in which its association with body size was validated and showed that the anthropometric variables explained over 3% of their total variability (Additional file 1: Figure S6).

**Table 2:** Metabolites significantly associated with each anthropometric variable in the discovery and replication sets

***N***	Metabolites	Association with:					
		BMI		WC		WHR	
		***β*** (95% CI)	p*	***β*** (95% CI)	p*	***β*** (95% CI)	p*
1	Asparagine	− 1.02 (− 1.25; − 0.79)	< .001	− 1.11 (− 1.38; − 0.84)	< .001	− 0.80 (− 1.15; − 0.45)	< .001
2	Glutamine	− 0.83 (− 1.05; − 0.60)	< .001	− 0.92 (− 1.18; − 0.65)	< .001	− 0.69 (− 1.03; − 0.35)	< .001
3	Glutamate	1.03 (0.83; 1.23)	< .001	1.08 (0.84; 1.32)	< .001	0.64 (0.33; 0.95)	< .001
4	Glycine	− 1.01 (− 1.24; − 0.79)	< .001	− 1.31 (− 1.57; − 1.04)	< .001	− 1.40 (− 1.74; − 1.05)	< .001
5	Isoleucine	1.04 (0.80; 1.27)	< .001	1.24 (0.95; 1.52)	< .001	1.03 (0.66; 1.39)	< .001
6	Serine	− 0.93 (− 1.15; − 0.71)	< .001	− 1.02 (− 1.29; − 0.76)	< .001	− 1.04 (− 1.38; − 0.69)	< .001
7	Valine	1.35 (1.12; 1.58)	< .001	1.49 (1.22; 1.77)	< .001	1.17 (0.82; 1.53)	< .001
8	LysoPC a C17:0	− 1.14 (− 1.35; − 0.92)	< .001	− 1.31 (− 1.56; − 1.05)	< .001	− 0.82 (− 1.15; − 0.48)	< .001
9	LysoPC a C18:1	−1.36 (− 1.57; − 1.16)	< .001	− 1.51 (− 1.75; − 1.27)	< .001	− 0.97 (− 1.28; − 0.65)	< .001
10	LysoPC a C18:2	− 1.47 (− 1.68; − 1.25)	< .001	− 1.59 (− 1.85; − 1.33)	< .001	− 1.00 (− 1.34; − 0.66)	< .001
11	PC aa C38:3	1.69 (1.46; 1.92)	< .001	1.85 (1.57; 2.12)	< .001	1.62 (1.27; 1.98)	< .001
12	PC aa C38:4	1.06 (0.83; 1.30)	< .001	1.26 (0.98; 1.54)	< .001	1.20 (0.84; 1.56)	< .001
13	PC aa C42:0	− 0.22 (− 0.27; − 0.17)	< .001	− 0.26 (− 0.32; − 0.20)	< .001	− 0.23 (− 0.31; − 0.15)	< .001
14	PC ae C34:3	− 1.26 (− 1.49; − 1.03)	< .001	− 1.7 (− 1.97; − 1.42)	< .001	− 1.77 (− 2.12; − 1.42)	< .001
15	PC ae C40:5	− 1.06 (− 1.29; − 0.83)	< .001	− 1.24 (− 1.51; − 0.97)	< .001	− 0.99 (− 1.34; − 0.64)	< .001
16	PC ae C42:5	− 1.13 (− 1.35; − 0.91)	< .001	− 1.34 (− 1.6; − 1.08)	< .001	− 1.14 (− 1.48; − 0.8)	< .001
17	Acylcarnitine C0	0.85 (0.61; 1.09)	< .001	0.92 (0.64; 1.21)	< .001	–	–
18	Acylcarnitine C3	0.27 (0.14; 0.41)	< .001	0.34 (0.18; 0.49)	< .001	–	–
19	Acylcarnitine C5	0.69 (0.51; 0.86)	< .001	0.63 (0.42; 0.83)	< .001	–	–
20	Leucine	1.09 (0.85; 1.33)	< .001	1.23 (0.95; 1.52)	< .001	–	–
21	Phenylalanine	0.78 (0.55; 1.01)	< .001	0.88 (0.61; 1.15)	< .001	–	–
22	Tyrosine	1.23 (0.98; 1.47)	< .001	1.22 (0.92; 1.51)	< .001	–	–
23	Kynurenine	1.10 (0.87; 1.33)	< .001	0.96 (0.68; 1.23)	< .001	–	–
24	PC aa C32:1	0.58 (0.36; 0.81)	< .001	0.46 (0.20; 0.73)	0.001	–	–
25	PC aa C34:4	0.57 (0.32; 0.81)	<. 001	0.44 (0.15; 0.73)	0.004	–	–
26	PC aa C38:0	− 0.46 (− 0.68; − 0.24)	<. 001	− 0.62 (− 0.88; − 0.36)	< .001	–	–
27	PC aa C40:2	− 0.11 (− 0.17; − 0.05)	< .001	− 0.14 (− 0.21; − 0.08)	< .001	–	–
28	PC aa C40:4	0.61 (0.36; 0.85)	< .001	0.78 (0.50; 1.07)	< .001	–	–
29	PC aa C40:6	0.73 (0.49; 0.96)	< .001	0.76 (0.49; 1.04)	< .001	–	–
30	PC aa C42:1	− 0.12 (− 0.15; − 0.09)	< .001	− 0.15 (− 0.18; − 0.11)	< .001	–	–
31	PC aa C42:2	− 0.56 (− 0.72; − 0.40)	< .001	− 0.61 (− 0.80; − 0.42)	< .001	–	–
32	PC ae C32:1	− 0.57 (− 0.78; − 0.37)	< .001	− 0.9 (− 1.14; − 0.66)	< .001	–	–
33	PC ae C34:2	− 0.92 (− 1.14; − 0.71)	< .001	− 1.24 (− 1.49; − 0.98)	< .001	–	–
34	PC ae C36:2	− 1.12 (− 1.35; − 0.90)	< .001	− 1.32 (− 1.59; − 1.05)	< .001	–	–
35	PC ae C36:3	−0.87 (− 1.10; − 0.65)	< .001	− 1.15 (− 1.42; − 0.88)	< .001	–	–
36	PC ae C38:2	− 1.05 (− 1.27; − 0.82)	< .001	− 1.19 (− 1.46; − 0.92)	< .001	–	–
37	PC ae C40:6	− 0.89 (− 1.12; − 0.65)	< .001	− 1.11 (− 1.38; − 0.83)	< .001	–	–
38	PC ae C44:4	− 1.05 (− 1.24; − 0.86)	< .001	− 1.11 (− 1.34; − 0.89)	< .001	–	–
39	SM C16:0	− 0.58 (− 0.74; − 0.42)	< .001	− 0.64 (− 0.83; − 0.45)	< .001	–	–
40	PC ae C44:6	− 0.64 (− 0.87; − 0.42)	< .001	–	–	–	–
41	SM C18:1	0.49 (0.34; 0.64)	< .001	–	–	–	–
42	Hexoses	0.65 (0.44; 0.87)	< .001	–	–	–	–
43	PC aa C32:2	–	–	0.45 (0.19; 0.72)	0.002	–	–

Analysis using residuals from Z and Log transformed metabolites with fixed effect for country and sex and random effect for batches nested within study. The multivariable model included additional adjustment for height, physical activity, smoking status, education level, alcohol consumption, dietary intakes of total energy, red and processed meats, fish and shellfish, and fibre, age at blood collection and fasting status. *P value refers to FDR correction

**Fig. 2:**
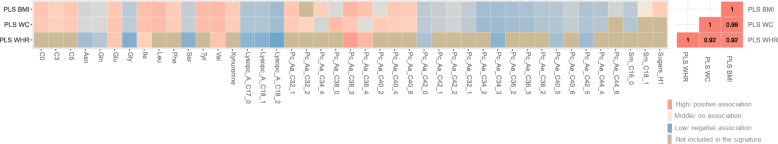
Pearson correlation between the PLS scores of BMI, WC, and WHR and their loadings

PLS regression analysis defined metabolic signatures of BMI, WC, and WHR (Fig. 2). The metabolites with the greatest contribution to the metabolic signature of BMI were PC aa C38:3 (loading = 0.19), valine (loading = 0.18), tyrosine (loading = 0.17), lysoPC C18:2 (loading = − 0.25), phosphatidylcholine acyl-alkyl (PC ae) C34:2 (loading = − 0.25), and PC ae C34:3 (loading = − 0.25). Similar results were observed for the metabolic signatures of the other anthropometric measures (Additional file 1: Table S3). In the replication set, the metabolic signatures of greater body sizes showed a moderate Pearson correlation with BMI (*r* = 0.48, 95% CI 0.44–0.53, *p* < 0.001), WC (*r* = 0.39, 95% CI 0.34–0.43, *p* < 0.001), and WHR (*r* = 0.28, 95% CI 0.24–0.33, *p* < 0.001). Similar correlations were found among CRC and endometrial cancer controls and for men and women (Additional file 1: Table S4). Additionally, the metabolic signatures of BMI, WC, and WHR explained ~ 23%, ~ 16%, and ~ 8% of the variability of their respective anthropometric variable, among CRC and endometrial cancer controls.

### Metabolic signatures of greater body size and cancer risk

**Fig. 3:**
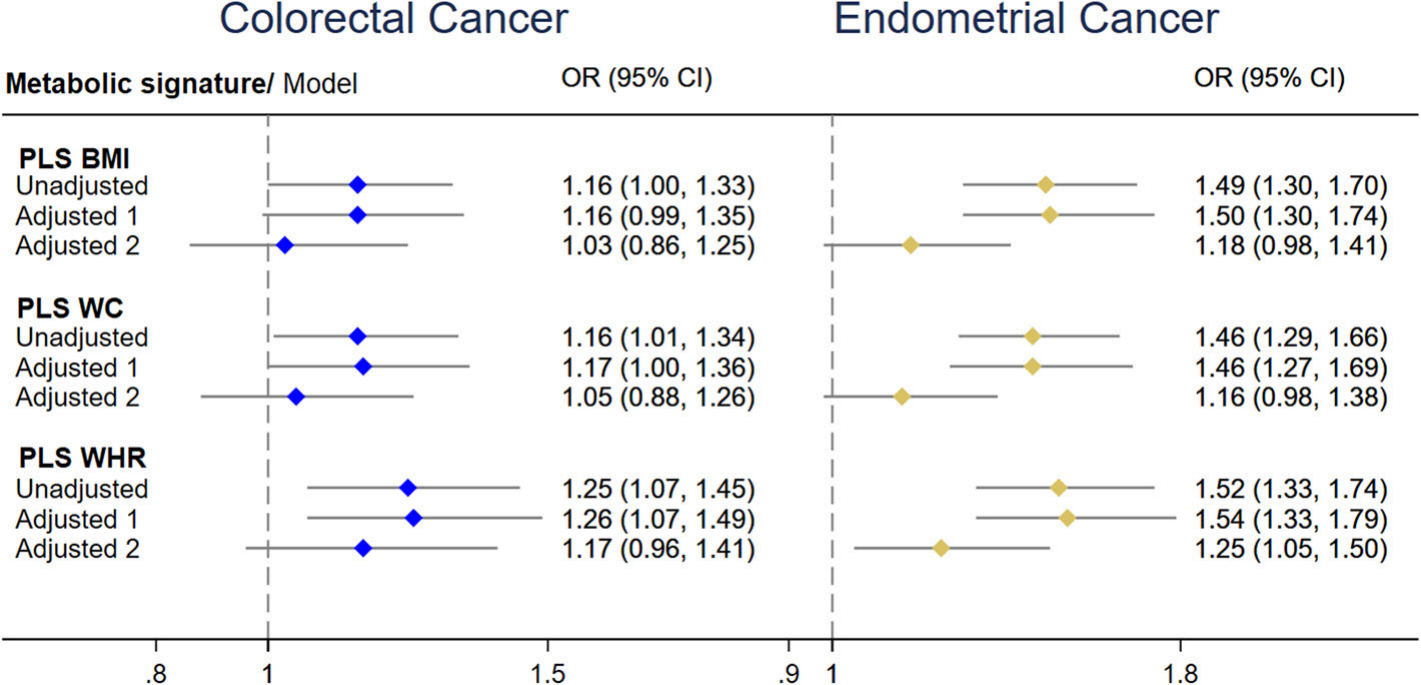
Association of colorectal and endometrial cancers with the metabolic signatures of obesity. ORs and 95% CIs by 1-SD change. Adjusted model 1 was adjusted for height, physical activity, smoking status, education level, consumption of alcohol, total energy, red and processed meats, fish and shellfish, age at blood collection, and fasting status. For endometrial cancer, model 1 was further adjusted for menopause status, hormonal therapy, oral contraceptive use, age at first menstrual period, and age at first full-term pregnancy, while for colorectal cancer, model 1 was further adjusted for fibre and calcium intake. Model 2 included the adjustments from model 1 plus anthropometric measures

As shown in Fig. 3, endometrial cancer was positively associated with the metabolic signature of BMI (odds ratio per 1-standard deviation [OR_1-sd_] 1.50, 95% CI 1.30–1.74), WC (OR_1-sd_ 1.46, 95% CI 1.27–1.69), and WHR (OR_1-sd_ 1.54, 95% CI 1.33–1.79) after adjustment for the main confounders. Similarly, colorectal cancer was positively associated with the metabolic signature of WHR (OR_1-sd_: 1.26, 95% CI 1.07–1.49), but borderline statistically significantly with the metabolic signatures of BMI (OR_1-sd_: 1.16, 95% CI 0.99–1.35) and WC (OR_1-sd_: 1.17, 95% CI 1.00–1.36). The association between endometrial cancer and the metabolomic signature of WHR remained statistically significant even after adjustment for anthropometric measures of obesity. We also tested the effect of further adjusting the models for C-peptide and cancer grade and staging; however, the results remained similar to those without these adjustments (Additional file 1: Table S5). Similar results were also found when analyses were repeated removing the cancer cases reported in the first two years of follow-up (results not shown).

### Metabolic signatures of greater body size and weight loss

**Fig. 4:**
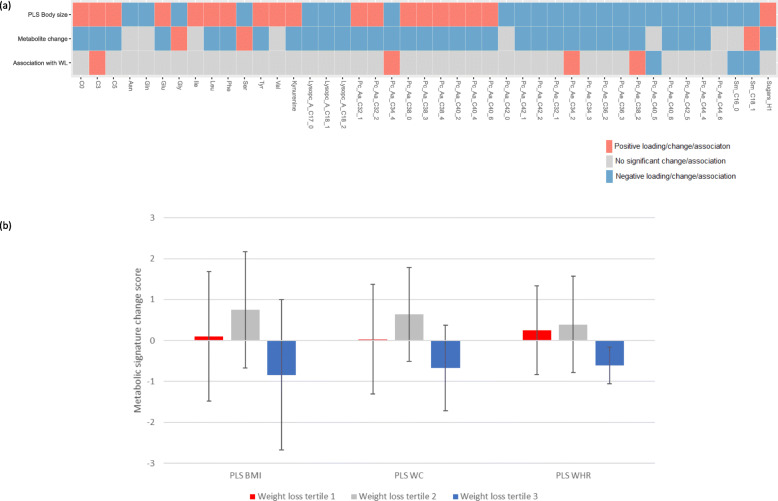
Metabolomics signatures of obesity, metabolite changes, and weight loss in the Intercept pilot intervention. **a** Comparison of the loadings for the metabolomics signatures of greater body sizes, changes in metabolites, and association between changes in metabolites and weight loss. **b** Means and standard deviations for metabolomics signatures of greater body sizes by tertiles of weight loss

The percentage of weight loss was positively correlated with changes in the metabolic signatures of BMI (*r* = 0.5, 95% CI 0.06–0.94, *p* = 0.03), WC (*r* = 0.5, 95% CI 0.05–0.94, *p* = 0.03), and WHR (*r* = 0.6, 95% CI 0.32–0.87, *p* = 0.01). Participants in the third tertile of weight loss (mean = 16.5 kg) showed the greatest reduction of the PLS scores for BMI, WC, and WHR compared to those in the first (mean = 10.6 kg) and second (mean = 12.7 kg) tertiles (Fig. 4). There were statistically significant changes in 105 measured metabolites during the intervention (Additional file 1: Table S6). As shown in Fig. 4, there were significant decreases in the serum levels of acylcarnitines C0, C3, C5, glutamate, leucine, phenylalanine, tyrosine, kynurenine, PCs aa C32:1, C32:2, C38:0, C38:3, C38:4, C40:2, C40:4, C40:6, and hexoses and an increase in the serum levels of glycine, serine, and sphingomyelin C18:1 following the intervention. All these changes were consistent with the metabolic signatures of greater body sizes. However, the association between changes in these metabolites and weight loss were mainly not significant (Additional file 1: Table S7 and Figure S7), which may reflect the small sample size (*N* = 17).

### Measured anthropometry and cancer risk

Analysis of the association between the three measures of obesity and cancer risk showed that greater BMI and WC were significantly associated with increased risk of endometrial and colorectal cancers, while greater WHR was only associated with increased risk of colorectal cancer (Additional file 1: Figure S8). When the models were further adjusted to their respective metabolic signature, all associations were attenuated, suggesting a partial mediating role of the metabolic signatures in these relationships (Additional file 1: Figure S8).

## Discussion

In this analysis which used data from a large prospective cohort, we identified metabolic signatures of BMI, WC, and WHR that were positively associated with colorectal and endometrial cancer risk. Further, the metabolomics signature of WHR predicted endometrial cancer risk beyond measured body fatness. In an exploratory analysis using data from a diet-induced weight loss intervention study, we found a positive association between weight loss and changes in the metabolic signatures of greater body sizes.

The metabolic signature of greater BMI, WC, and WHR was represented by higher levels of valine, isoleucine, glutamate, PC aa C38:3, PC aa C38:4 and lower levels of asparagine, glutamine, glycine, serine, lysoPC C17:0, lysoPC C18:1 and lysoPC C18:2, PC aa C42:0, PC ae C34:3, PC ae C40:5, and PC ae C42:5. Other metabolites were also shown to be relevant for the metabolic signatures of BMI and WC only, including leucine, phenylalanine, tyrosine, kynurenine, C0, C3, C5, SM C16:0, SM C18:1, and 17 glycerophospholipids. Additionally, many of these metabolites changed significantly after the Intercept weight loss intervention, reinforcing their association with body weight.

In line with previous studies, the current analysis showed associations between greater body size and the amino acids valine, leucine, isoleucine, tyrosine, glutamate, and the biogenic amine kynurenine [10, 11, 13]. However, this study also provided strong evidence for the associations between body weight and the amino acids phenylalanine, asparagine, glutamine, and glycine in EPIC, which has been supported by other metabolomics studies [30–32]. Systematic reviews of case-control studies examining the association of serum concentrations of metabolites and cancer diagnosis reported that tyrosine and phenylalanine are associated with both colorectal and endometrial cancers, and valine and glutamate with endometrial cancer only [33, 34]. These amino acids have been reported to be associated with insulin resistance and impaired insulin secretion [35], key factors of cancer and obesity pathogenesis [36, 37]. Valine, as well as other branched chain amino acids (BCAAs), also plays an important role in activating the mechanistic/mammalian target of rapamycin (mTOR) axis, a signalling pathway associated with cell growth, proliferation, and survival [38], important features of cancer development. Imbalances in the biogenic amine kynurenine metabolism have also been implicated in cancer development [39, 40].

Consistent with other studies, the metabolic signature of obesity reflected lipid dysregulation [10, 11, 30, 41, 42], such as higher levels of diacyl PCs and lower levels of acyl-alkyl PCs and lysoPCs. Some of these lipid alterations have also been associated with cancer risk, for example, LysoPC C18:1 and C18:2 have been reported to be downregulated in colorectal cancer patients [43]. LysoPCs are important cell-signalling molecules and their downregulation may reflect pathophysiological changes in cancer development [43]. Recent prospective studies have also reported lower levels of PC ae C34:2, C36:2, C36:3, and C38:2 to be associated with breast cancer risk [8]. The acyl-alkyl PCs seem to have antioxidant properties and when downregulated may increase reactive oxygen species (ROS) generation, promoting oxidative stress and oncogenic DNA defects [44]. Increased ROS has also been linked to many metabolic alterations, such as insulin resistance [45], decreases in adiponectin, and increased expression of pro-inflammatory cytokines including TNFα and IL-6 [46], all potential markers of obesity and cancer development. Regarding the alterations in diacyl PCs, the metabolites PC aa 32:1 and PC aa C38:3 are of particular interest as they have been associated with diabetes and cardiovascular diseases [47, 48], although not yet to cancer. The role of these lipid metabolites is still not clear and remains to be further investigated.

The metabolic signature of greater WHR included metabolites that were also significantly associated with BMI and WC and may reflect overall greater body size. Importantly, it was positively associated with endometrial cancer risk regardless of the individual’s body fatness. This suggests the metabolic signature of WHR was potentially able to differentiate individuals with similar body size but different metabolic health status. These results corroborate other research studies indicating that metabolic alterations which typically accompany obesity, such as insulin resistance and hyperinsulinemia, may be more relevant risk factors for some cancers than adiposity per se [49–53].

Exploratory analysis of data from the Intercept study showed that changes in the metabolic signatures of greater body sizes were positively correlated with the percentage of weight loss. Consistent with previous intervention studies [31, 54], the Intercept weight loss intervention promoted reductions in levels of amino acids and biogenic amines that have been consistently positively associated with obesity and cancer risk, such as tyrosine, phenylalanine, glutamate, and kynurenine, suggesting reduction in cancer risk in individuals with obesity who lose weight. The intervention also decreased levels of PC aa C38:3, PC aa C38:4, and increased levels of glycine and serine, metabolites that have been strongly associated with greater body sizes and that may also be potentially linked to cancer risk. In support of this, primary results from Intercept showed that weight loss in individuals with obesity was associated with improvement in insulin levels and reduction in Ki-67 expression in colorectal tissue, an established marker of cell proliferation [16].

To our knowledge, this is one of the largest studies examining the associations of greater body size and metabolic profiles, and the first to relate these signatures to colorectal and endometrial cancer risk. Strengths of this study include the assessment of numerous behavioural factors and anthropometric measures in EPIC, allowing us to conduct a comprehensive analysis of the associations between greater body sizes and metabolites and to control for potential confounding factors. Additionally, in the current study, we were able to validate the association of metabolites and greater body sizes in a replication set. The analyses of the association of changes in the metabolic signatures of greater body sizes and weight loss in the Intercept intervention study are also novel. However, since anthropometric variables have been previously associated to cancer risk in EPIC, an external validation of the metabolic signatures is needed. Differences in sex distribution and fasting status between the samples may have also affected the results. Another potential limitation of this study was the use of targeted metabolomics data only, in which a set of metabolites that are known a priori are measured. Untargeted metabolomics could provide a more comprehensive view of the metabolic perturbations associated with obesity and cancer; however, such data are currently not available for this cohort. Nevertheless, the findings from the current targeted analysis provide some interesting clues as to the specific metabolic perturbations that accompany obesity and that could be associated with cancer development. The small sample size and exploratory nature of the Intercept intervention study are also limitations. Additionally, the Intercept sample was composed mainly of women, younger, and relatively healthy participants with obesity, and it lacked a control group and physical activity measures. Therefore, replication of the results of this study within a large randomised controlled trial is needed. In addition, the EPIC colorectal case-control sample was relatively small, limiting the possibilities of conducting stratified analysis by sex and cancer subsite. An additional limitation could be that metabolites were assayed in plasma samples of EPIC participants and but in serum samples of Intercept participants. Furthermore, despite the prospective design of EPIC and Intercept, we cannot rule out potential reverse causation since some metabolites may have been altered by underlying subclinical carcinogenic processes. To tackle this issue, we conducted sensitivity analyses excluding cases recorded in the first 2 years of follow-up, and similar results were obtained. Finally, although we excluded participants with diagnosed diabetes, we lacked data on other comorbidities such as hypertension and cardiovascular disease which may have impacted the assayed metabolic pathways and which might represent potential confounding factors. Future studies should aim to better understand the impact of such comorbidities on the metabolic disturbances underlying the association between obesity and cancer.

## Conclusions

Obesity is associated with a distinct metabolic signature comprising changes in levels of specific amino acids and lipids which is positively associated with both colorectal and endometrial cancer and is potentially reversible following weight loss. These findings may offer insights into the pathophysiological mechanisms underlying the obesity-cancer relation. Further, by measuring a specific panel of metabolites, it may be possible to identify strata of the population at higher risk for obesity-related cancers. Future studies should aim to further explore the impact of obesity on the metabolome using, for example, untargeted metabolomics which could uncover additional pathways that may be relevant for cancer development.

## Supplementary Information

**Additional file 1: Supplementary information**. **Table S1**. Number of metabolites included in each study population; **Table S2**. Metabolites associated with each anthropometric variable in the replication set; **Table S3**. Metabolites with the greatest contribution for each metabolic signature; **Table S4**. Correlations between the metabolic signatures and their corresponding anthropometric measure; **Table S5**. Metabolites that changed significantly from baseline to follow-up in the Intercept; **Table S6**. Metabolites significantly associated with weight loss in the Intercept; **Table S7**. Metabolites significantly associated with weight loss in the Intercept; **Figure S1**. Overall *R*_partial_2 and weighted *R*_partial_2 for each covariate and BMI in the discovery set; **Figure S2**. Overall *R*_partial_2 and weighted *R*_partial_2 for each covariate and WC in the discovery set; **Figure S3**. Overall *R*_partial_2 and weighted *R*_partial_2 for each covariate and WHR in the discovery set; **Figure S4**. Overall *R*_partial_2 and weighted *R*_partial_2 for each covariate and weight loss in the Intercept; **Figure S5**. Association of colorectal and endometrial cancers with the metabolic signatures further adjusted to C-peptide; **Figure S6**. Association of colorectal and endometrial cancers with the anthropometric measures of obesity; **Figure S7**. Smile plot of the associations between metabolites with weight loss in the Intercept.

## Data Availability

EPIC data and biospecimens are available for investigators who seek to answer important questions on health and disease in the context of research projects that are consistent with the legal and ethical standard practices of IARC/WHO and the EPIC Centres. The primary responsibility for accessing the data belongs to IARC and the EPIC centres. Access to materials from the EPIC study can be requested by contacting epic@iarc.fr.

## Abbreviations

BMI: Body mass index
EPIC: European Prospective Investigation into Cancer and Nutrition
HCC: Hepatocellular carcinoma risk
LOD: Limit of detection
LOQ: Limit of quantification
LysoPC: Lysophosphatidylcholine acyl
PC aa: Phosphatidylcholine diacyl
PC ae: Phosphatidylcholine acyl-alkyl
WC: Waist circumference
WHR: Waist-to-hip ratio
