# Supplementary material for: Metabolic signatures of greater body size and their associations with risk of colorectal and endometrial cancers in the European Prospective Investigation into Cancer and Nutrition
Source: BMC Med. 2021 Apr 30;19:101. doi: 10.1186/s12916-021-01970-1 (PMC8086283; doi:10.1186/s12916-021-01970-1)
Supplement: Supplementary file 1 — Additional file 1: Supplementary information. Table S1. Number of metabolites included in each study population; Table S2. Metabolites associated with each anthropometric variable in the replication set; Table S3. Metabolites with the greatest contribution for each metabolic signature; Table S4. Correlations between the metabolic signatures and their corresponding anthropometric measure; Table S5. Metabolites that changed significantly from baseline to follow-up in the Intercept; Table S6. Metabolites significantly associated with weight loss in the Intercept; Table S7. Metabolites significantly associated with weight loss in the Intercept; Figure S1. Overall Rpartial2 and weighted Rpartial2 for each covariate and BMI in the discovery set; Figure S2. Overall Rpartial2 and weighted Rpartial2 for each covariate and WC in the discovery set; Figure S3. Overall Rpartial2 and weighted Rpartial2 for each covariate and WHR in the discovery set; Figure S4. Overall Rpartial2 and weighted Rpartial2 for each covariate and weight loss in the Intercept; Figure S5. Association of colorectal and endometrial cancers with the metabolic signatures further adjusted to C-peptide; Figure S6. Association of colorectal and endometrial cancers with the anthropometric measures of obesity; Figure S7. Smile plot of the associations between metabolites with weight loss in the Intercept. [file 12916_2021_1970_MOESM1_ESM.docx]

**Manuscript: Metabolic signatures of greater body size and their associations with risk of colorectal and endometrial cancers in the European Prospective Investigation into Cancer and Nutrition**

**Additional file 1:**

Table S1. Number of metabolites included in each study population…………………………..………………………2

Table S2. Metabolites associated with each anthropometric variable in the replication set…………..……3

Table S3. Metabolites with the greatest contribution for each metabolic signature ……………….………..…4

Table S4. Correlations between the metabolic signatures and their corresponding anthropometric measure ……………………………………………………………………………………………………………………………………………4

Table S5. Association of colorectal and endometrial cancers with the metabolic signatures with further adjustments to C-peptide, cancer stage and grade……………………………………………………………………………………………5

Table S6. Metabolites that changed significantly from baseline to follow-up in the Intercept………..…….6

Table S7. Metabolites significantly associated with weight loss in the Intercept…….………………….………7

Figure S1. Overall *R*_partial_2 and weighted *R*_partial_2 for each covariate and BMI in the discovery set……….8

Figure S2. Overall *R*_partial_2 and weighted *R*_partial_2 for each covariate and WC in the discovery set..…….9

Figure S3. Overall *R*_partial_2 and weighted *R*_partial_2 for each covariate and WHR in the discovery set…...10

Figure S4. Overall *R*_partial_2 and weighted *R*_partial_2 for each covariate and weight loss in the Intercept..11

Figure S5. Flow-diagram with the main methodological steps of the study……………………………………..12

Figure S6. Overall *R*_partial_2 and weighted *R*_partial_2 for residuals of metabolites that had their association with markers of obesity validated and anthropometric variables in the discovery set……………………..13

Figure S7. Smile plot of the associations between metabolites with weight loss in the Intercept…….15

Figure S8 Association of colorectal and endometrial cancers with the anthropometric measures of obesity…16

**Table S1. Number of metabolites included in each study population.**

| **Metabolites** | **European Prospective Investigation into Cancer and Nutrition** | | | | | | | | |  | **Intercept** |
| --- | --- | --- | --- | --- | --- | --- | --- | --- | --- | --- | --- |
|  | Discovery |  | Replication |  | Colorectal  case-control |  | | Endometrial  case-control |  |  |  |
|  | N | | N | | N | | N | | |  | N |
| Acylcarnitines | 14 | | 14 | | 14 | | 12 | | |  | 13 |
| Amino acids | 21 | | 21 | | 21 | | 21 | | |  | 21 |
| Biogenic amines | 6 | | 6 | | 6 | | 6 | | |  | 6 |
| Glycerophospholipids | 75 | | 75 | | 75 | | 74 | | |  | 75 |
| Sphingolipids | 12 | | 12 | | 12 | | 10 | | |  | 12 |
| Hexoses | 1 | | 1 | | 1 | | 1 | | |  | 1 |
| **Total** | **129** | | **129** | | **129** | | **124** | | |  | **128** |

**Table S2. Metabolites associated with each anthropometric variable in the replication set.**

| **N** | **Metabolites** | **Association with:** | | | | | |  |
| --- | --- | --- | --- | --- | --- | --- | --- | --- |
|  |  | **BMI** |  | **WC** |  | **WHR** |  |  |
|  |  | **β(95%CI)** | ***p*** | **β(95%CI)** | ***p*** | **β(95%CI)** | ***p*** |  |
| 1 | Asparagine | -0.98 (-1.33; -0.63) | <.001 | -1.12 (-1.54; -0.71) | <.001 | -1.15 (-1.71; -0.58) | 0.010 |  |
| 2 | Glutamine | -0.75 (-1.05; -0.44) | <.001 | -0.96 (-1.31; -0.6) | <.001 | -1.2 (-1.68; -0.72) | <.001 |  |
| 3 | Glutamate | 1.22 (0.9; 1.53) | <.001 | 1.44 (1.07; 1.81) | <.001 | 1.18 (0.67; 1.69) | 0.001 |  |
| 4 | Glycine | -1.22 (-1.56; -0.88) | <.001 | -1.62 (-2.02; -1.21) | <.001 | -1.69 (-2.24; -1.14) | <.001 |  |
| 5 | Isoleucine | 1.14 (0.78; 1.51) | <.001 | 1.38 (0.95; 1.81) | <.001 | 1.24 (0.65; 1.82) | 0.005 |  |
| 6 | Serine | -1.02 (-1.35; -0.68) | <.001 | -1.15 (-1.55; -0.75) | <.001 | -1.09 (-1.64; -0.55) | 0.011 |  |
| 7 | Valine | 1.4 (1.07; 1.74) | <.001 | 1.54 (1.14; 1.94) | <.001 | 1.33 (0.78; 1.87) | <.001 |  |
| 8 | LysoPC a C17:0 | -0.92 (-1.22; -0.62) | <.001 | -1.07 (-1.43; -0.71) | <.001 | -0.91 (-1.4; -0.42) | 0.035 |  |
| 9 | LysoPC a C18:1 | -1.23 (-1.53; -0.94) | <.001 | -1.45 (-1.8; -1.1) | <.001 | -1.39 (-1.87; -0.91) | <.001 |  |
| 10 | LysoPC a C18:2 | -1.57 (-1.9; -1.25) | <.001 | -1.58 (-1.96; -1.19) | <.001 | -1.29 (-1.82; -0.75) | <.001 |  |
| 11 | PC aa C38:3 | 1.79 (1.45; 2.14) | <.001 | 2.11 (1.7; 2.52) | <.001 | 2.09 (1.53; 2.65) | <.001 |  |
| 12 | PC aa C38:4 | 1.05 (0.7; 1.4) | <.001 | 1.31 (0.9; 1.72) | <.001 | 1.25 (0.69; 1.8) | 0.002 |  |
| 13 | PC aa C42:0 | -0.24 (-0.31; -0.16) | <.001 | -0.26 (-0.35; -0.17) | <.001 | -0.22 (-0.34; -0.1) | 0.030 |  |
| 14 | PC ae C34:3 | -1.19 (-1.52; -0.86) | <.001 | -1.27 (-1.66; -0.88) | <.001 | -1.11 (-1.65; -0.58) | 0.006 |  |
| 15 | PC ae C40:5 | -1.05 (-1.38; -0.71) | <.001 | -1.17 (-1.56; -0.77) | <.001 | -1.1 (-1.64; -0.56) | 0.008 |  |
| 16 | PC ae C42:5 | -1.25 (-1.58; -0.92) | <.001 | -1.47 (-1.86; -1.09) | <.001 | -1.42 (-1.95; -0.89) | <.001 |  |
| 17 | Acylcarnitine C0 | 0.94 (0.57; 1.3) | <.001 | 0.94 (0.51; 1.38) | 0.003 | - | - |  |
| 18 | Acylcarnitine C3 | 0.38 (0.17; 0.58) | 0.044 | 0.46 (0.22; 0.71) | 0.023 | - | - |  |
| 19 | Acylcarnitine C5 | 0.63 (0.38; 0.88) | <.001 | 0.68 (0.38; 0.97) | 0.001 | - | - |  |
| 20 | Leucine | 1.08 (0.72; 1.43) | <.001 | 1.2 (0.77; 1.62) | <.001 | - | - |  |
| 21 | Phenylalanine | 0.72 (0.38; 1.07) | 0.006 | 0.91 (0.5; 1.32) | 0.002 | - | - |  |
| 22 | Tyrosine | 1.26 (0.91; 1.62) | <.001 | 1.45 (1.03; 1.87) | <.001 | - | - |  |
| 23 | Kynurenine | 1.06 (0.73; 1.4) | <.001 | 1.08 (0.68; 1.47) | <.001 | - | - |  |
| 24 | PC aa C32:1 | 0.73 (0.39; 1.07) | 0.004 | 0.77 (0.37; 1.17) | 0.025 | - | - |  |
| 25 | PC aa C34:4 | 0.73 (0.38; 1.07) | 0.004 | 0.95 (0.55; 1.36) | 0.001 | - | - |  |
| 26 | PC aa C38:0 | -0.65 (-0.97; -0.33) | 0.007 | -0.75 (-1.12; -0.38) | 0.011 | - | - |  |
| 27 | PC aa C40:2 | -0.16 (-0.24; -0.08) | 0.018 | -0.18 (-0.28; -0.09) | 0.032 | - | - |  |
| 28 | PC aa C40:4 | 0.77 (0.41; 1.13) | 0.004 | 0.98 (0.56; 1.4) | 0.001 | - | - |  |
| 29 | PC aa C40:6 | 0.66 (0.32; 1) | 0.016 | 0.82 (0.42; 1.21) | 0.008 | - | - |  |
| 30 | PC aa C42:1 | -0.16 (-0.2; -0.11) | <.001 | -0.16 (-0.22; -0.11) | <.001 | - | - |  |
| 31 | PC aa C42:2 | -0.65 (-0.89; -0.42) | <.001 | -0.72 (-1; -0.44) | <.001 | - | - |  |
| 32 | PC ae C32:1 | -0.55 (-0.84; -0.25) | 0.034 | -0.66 (-1.01; -0.31) | 0.026 | - | - |  |
| 33 | PC ae C34:2 | -0.94 (-1.25; -0.62) | <.001 | -0.91 (-1.29; -0.54) | <.001 | - | - |  |
| 34 | PC ae C36:2 | -1.05 (-1.38; -0.71) | <.001 | -0.97 (-1.36; -0.57) | <.001 | - | - |  |
| 35 | PC ae C36:3 | -0.8 (-1.13; -0.47) | <.001 | -0.78 (-1.17; -0.39) | 0.012 | - | - |  |
| 36 | PC ae C38:2 | -0.92 (-1.27; -0.57) | <.001 | -0.83 (-1.24; -0.42) | 0.012 | - | - |  |
| 37 | PC ae C40:6 | -1.02 (-1.37; -0.68) | <.001 | -1.12 (-1.53; -0.71) | <.001 | - | - |  |
| 38 | PC ae C44:4 | -0.75 (-1.03; -0.47) | <.001 | -0.86 (-1.2; -0.53) | <.001 | - | - |  |
| 39 | SM C16:0 | -0.56 (-0.8; -0.32) | 0.001 | -0.65 (-0.94; -0.37) | 0.001 | - | - |  |
| 40 | PC ae C44:6 | -0.63 (-0.97; -0.3) | 0.031 | - | - | - | - |  |
| 41 | SM C18:1 | 0.43 (0.2; 0.66) | 0.031 | - | - | - | - |  |
| 42 | Hexoses | 0.62 (0.31; 0.92) | 0.012 | - | - | - | - |  |
| 43 | PC aa C32:2 | - | - | 0.78 (0.37; 1.18) | 0.026 | - | - |  |

**Note.** Analysis using residuals from Z and Log transformed metabolites with fixed effect for country and sex and random effect for batches. The multivariable model included additional adjustment for height, physical activity, smoking status, education level, alcohol consumption, dietary intakes of total energy, red and processed meats, fish and shellfish, and fibre, age at blood collection and fasting status.

**Table S3. Metabolites with the greatest contribution for each metabolic signature.**

| **Metabolite** | **PLS BMI** | **PLS WC** | **PLS WHR** |
| --- | --- | --- | --- |
|  | Loading | Loading | Loading |
| LysoPC a C18:2 | -0.255 | -0.251 | -0.380 |
| PC ae C34:3 | -0.248 | -0.261 |  |
| PC ae C34:2 | -0.246 | -0.263 |  |
| Glycine |  |  | -0.329 |
| Serine |  |  | -0.321 |
| Tyrosine | 0.172 | 0.167 |  |
| Valine | 0.184 | 0.176 | 0.143 |
| PC aa C38:3 | 0.192 |  | 0.275 |
| PC aa C38:0 |  | 0.169 |  |
| PC aa C38:4 |  |  | 0.188 |

**Table S4. Pearson correlations between the metabolic signatures and their corresponding anthropometric measure.**

|  | **Pearson correlation (r (95%CI)) between:** | | | | |
| --- | --- | --- | --- | --- | --- |
|  | PLS BMI and BMI | | PLS WC and WC | PLS WHR and WHR | |
| **Sample** |  |  | | |  |
| Replication, all | 0.48 (0.44 – 0.53) | 0.39 (0.34 – 0.43) | | | 0.28 (0.24 – 0.33) |
| Replication, men | 0.47 (0.42 – 0.53) | 0.44 (0.38 – 0.50) | | | 0.41 (0.35 – 0.42) |
| Replication, women | 0.51 (0.44 – 0.58) | 0.51 (0.44 – 0.59) | | | 0.39 (0.29 – 0.49) |
| Colorectal controls | 0.47 (0.40 – 0.54) | 0.37 (0.29 – 0.46) | | | 0.24 (0.16 – 0.33) |
| Endometrial, controls | 0.48 (0.41 – 0.54) | 0.48 (0.42 – 0.54) | | | 0.40 (0.34 – 0.46) |

**Table S5. Association of colorectal and endometrial cancers with the metabolic signatures with further adjustments to C-peptide, cancer stage and grade**

| **Metabolic signature/ Model** | **Colorectal cancer** | **Endometrial Cancer** |
| --- | --- | --- |
|  | OR (95%CI) | OR (95%CI) |
| **Models further adjusted to C-peptide** |  |  |
| BMI |  |  |
| Adjusted 1 | 1.21 (1.02; 1.42) | 1.49 (1.28; 1.73) |
| Adjusted 2 | 1.06 (0.88; 1.29) | 1.18 (0.98; 1.41) |
| WC |  |  |
| Adjusted 1 | 1.21 (1.03; 1.43) | 1.45 (1.25; 1.68) |
| Adjusted 2 | 1.08 (0.90; 1.31) | 1.16 (0.97; 1.38) |
| WHR |  |  |
| Adjusted 1 | 1.33 (1.12; 1.58) | 1.53 (1.31; 1.78) |
| Adjusted 2 | 1.21 (0.99; 1.48) | 1.25 (1.05; 1.50) |
| **Models further adjusted to cancer stage** |  |  |
| BMI |  |  |
| Adjusted 1 | 1.08 (0.86; 1.33) | 1.53 (1.28; 1.83) |
| Adjusted 2 | 1.01 (0.77; 1.31) | 1.06 (0.84; 1.34) |
| WC |  |  |
| Adjusted 1 | 1.08 (0.88; 1.34) | 1.48 (1.24; 1.77) |
| Adjusted 2 | 1.03 (0.79; 1.33) | 1.05 (0.84; 1.31) |
| WHR |  |  |
| Adjusted 1 | 1.26 (1.07; 1.48) | 1.58 (1.31; 1.90) |
| Adjusted 2 | 1.12 (0.85; 1.46) | 1.17 (0.93; 1.47) |
| **Models further adjusted to cancer grade** |  |  |
| BMI |  |  |
| Adjusted 1 | 1.15 (0.98; 1.35) | 2.16 (1.37; 3.39) |
| Adjusted 2 | 1.03 (0.85; 1.25) | 1.22 (0.68; 2.18) |
| WC |  |  |
| Adjusted 1 | 1.16 (0.99; 1.36) | 2.28 (1.42; 3.65) |
| Adjusted 2 | 1.05 (0.87; 1.26) | 1.28 (0.70; 2.34) |
| WHR |  |  |
| Adjusted 1 | 1.26 (1.07; 1.49) | 2.19 (1.40; 3.43) |
| Adjusted 2 | 1.17 (0.96; 1.43) | 1.42 (0.83; 2.43) |

**Note.** Model 1 was adjusted for height, physical activity, smoking status, education level, consumption of alcohol, total energy, red and processed meats, fish and shellfish, age at blood collection, fasting status and the further adjustment for C-peptide or cancer grade or cancer stage. For endometrial cancer model 1 was further adjusted to menopause status, hormonal therapy, oral contraceptive use, age at first menstrual period and age at first full pregnancy, while for colorectal cancer model 1 was further adjusted for fibre and calcium intake. Model 2 included the adjustments from model 1 plus anthropometric measures.

**Table S6. Metabolites that changed significantly from baseline to follow-up in the Intercept.**

| Metabolites | N | Baseline | | Follow-up | | Difference | | T-test | P-value* |
| --- | --- | --- | --- | --- | --- | --- | --- | --- | --- |
|  |  | Mean | SD | Mean | SD | Mean | SE |  |  |
| C0 | 17 | 36.54 | 7.69 | 29.11 | 8.09 | -7.43 | 1.37 | 5.44 | <.001 |
| C12 | 17 | 0.08 | 0.04 | 0.11 | 0.04 | 0.04 | 0.01 | -2.81 | 0.01 |
| C14:1 | 17 | 0.07 | 0.02 | 0.13 | 0.03 | 0.06 | 0.01 | -5.25 | <.001 |
| C14:2 | 17 | 0.03 | 0.02 | 0.05 | 0.01 | 0.02 | 0 | -5.11 | <.001 |
| C16 | 17 | 0.11 | 0.02 | 0.14 | 0.03 | 0.03 | 0.01 | -3.85 | <.001 |
| C18:1 | 17 | 0.1 | 0.02 | 0.16 | 0.03 | 0.05 | 0.01 | -7.26 | <.001 |
| C18:2 | 17 | 0.04 | 0.02 | 0.05 | 0.02 | 0.01 | 0 | -2.26 | 0.04 |
| C2 | 17 | 5.33 | 1.32 | 8.68 | 2 | 3.35 | 0.58 | -5.76 | <.001 |
| C3 | 17 | 0.32 | 0.11 | 0.22 | 0.09 | -0.1 | 0.02 | 6.23 | <.001 |
| C4 | 17 | 0.17 | 0.05 | 0.15 | 0.05 | -0.02 | 0.01 | 3.06 | 0.01 |
| C5 | 17 | 0.12 | 0.05 | 0.09 | 0.03 | -0.03 | 0.01 | 4.38 | <.001 |
| Arginine | 17 | 114.36 | 22.59 | 99.97 | 19.62 | -14.39 | 4.81 | 2.99 | 0.01 |
| Citrulline | 17 | 30.09 | 4.76 | 24.22 | 5.25 | -5.87 | 1.29 | 4.56 | <.001 |
| Glutamate | 17 | 50.84 | 15.81 | 36.01 | 14.63 | -14.83 | 2.95 | 5.03 | <.001 |
| Glycine | 17 | 225.88 | 61.91 | 253.53 | 48.58 | 27.65 | 9.2 | -3.01 | 0.01 |
| Histidine | 17 | 82.52 | 9.96 | 73.91 | 11.14 | -8.62 | 2.83 | 3.04 | 0.01 |
| Leucine | 17 | 127.74 | 24.29 | 112.48 | 28.55 | -15.26 | 5.87 | 2.6 | 0.01 |
| Methionine | 17 | 22.78 | 3.72 | 20.72 | 3.77 | -2.05 | 0.94 | 2.19 | 0.05 |
| Ornithine | 17 | 58.76 | 14.6 | 46.18 | 7.75 | -12.58 | 3.64 | 3.45 | <.001 |
| Phenylalanine | 17 | 64.25 | 8.54 | 55.37 | 9.34 | -8.88 | 2.24 | 3.97 | <.001 |
| Proline | 17 | 175.48 | 58.52 | 148.26 | 36.46 | -27.22 | 10.27 | 2.65 | 0.01 |
| Serine | 17 | 108.04 | 16.41 | 121.79 | 14.37 | 13.75 | 4.34 | -3.17 | <.001 |
| Thryptophane | 17 | 66.55 | 13.6 | 50.79 | 13.4 | -15.76 | 3.56 | 4.43 | <.001 |
| Tyrosine | 17 | 69.19 | 17 | 54.71 | 14.62 | -14.48 | 4.08 | 3.55 | <.001 |
| ADMA | 17 | 0.46 | 0.07 | 0.4 | 0.05 | -0.06 | 0.02 | 3.74 | <.001 |
| Kynurenine | 17 | 2.81 | 0.69 | 2.01 | 0.34 | -0.81 | 0.17 | 4.66 | <.001 |
| Sarcosine | 17 | 1.34 | 0.61 | 0.96 | 0.49 | -0.38 | 0.14 | 2.66 | 0.02 |
| t4-OH-Pro | 17 | 9.71 | 3.77 | 6.16 | 1.77 | -3.55 | 1.04 | 3.4 | <.001 |
| lysoPC a C16:1 | 17 | 3.14 | 1.09 | 2.07 | 0.74 | -1.06 | 0.24 | 4.49 | <.001 |
| lysoPC a C17:0 | 17 | 1.84 | 0.49 | 1.36 | 0.48 | -0.48 | 0.12 | 3.96 | <.001 |
| lysoPC a C18:0 | 17 | 29.06 | 7.44 | 19.06 | 6.94 | -10 | 2.15 | 4.65 | <.001 |
| lysoPC a C18:1 | 17 | 21.78 | 6.5 | 18.19 | 6.7 | -3.6 | 1.42 | 2.53 | 0.02 |
| lysoPC a C18:2 | 17 | 32.99 | 8.2 | 23.87 | 9.78 | -9.13 | 2.52 | 3.63 | <.001 |
| lysoPC a C20:3 | 17 | 2.24 | 0.54 | 1.22 | 0.71 | -1.01 | 0.16 | 6.21 | <.001 |
| PC aa C28:1 | 17 | 3.15 | 0.7 | 2.08 | 0.46 | -1.07 | 0.11 | 9.36 | <.001 |
| PC aa C30:0 | 17 | 5.07 | 1.96 | 2.39 | 0.72 | -2.69 | 0.41 | 6.55 | <.001 |
| PC aa C32:0 | 17 | 17.32 | 3.28 | 15.97 | 3.47 | -1.36 | 0.61 | 2.21 | 0.05 |
| PC aa C32:1 | 17 | 24.15 | 10.64 | 12.41 | 6.01 | -11.74 | 2.13 | 5.52 | <.001 |
| PC aa C32:2 | 17 | 6.93 | 2.18 | 1.97 | 1.23 | -4.96 | 0.62 | 7.99 | <.001 |
| PC aa C32:3 | 17 | 0.75 | 0.17 | 0.47 | 0.08 | -0.28 | 0.03 | 8.23 | <.001 |
| PC aa C34:1 | 17 | 213.18 | 33.49 | 184.06 | 31.6 | -29.12 | 5.93 | 4.91 | <.001 |
| PC aa C34:2 | 17 | 325.71 | 19.43 | 287.18 | 32.47 | -38.53 | 7.55 | 5.11 | <.001 |
| PC aa C34:3 | 17 | 23.11 | 6.49 | 9.92 | 4.35 | -13.19 | 1.77 | 7.45 | <.001 |
| PC aa C34:4 | 17 | 2.61 | 0.73 | 0.81 | 0.39 | -1.81 | 0.2 | 9.08 | <.001 |
| PC aa C36:0 | 17 | 2.21 | 0.99 | 1.6 | 0.63 | -0.61 | 0.11 | 5.46 | <.001 |
| PC aa C36:1 | 17 | 57.98 | 16 | 28.97 | 8.93 | -29.01 | 3.63 | 7.99 | <.001 |
| PC aa C36:2 | 17 | 230 | 16.47 | 149.21 | 35.97 | -80.79 | 9.12 | 8.86 | <.001 |
| PC aa C36:3 | 17 | 133.12 | 21.16 | 82.59 | 28.18 | -50.53 | 4.86 | 10.4 | <.001 |
| PC aa C36:5 | 17 | 35.96 | 15.18 | 17.54 | 8.13 | -18.42 | 3.68 | 5 | <.001 |
| PC aa C36:6 | 17 | 1.3 | 0.48 | 0.46 | 0.23 | -0.84 | 0.11 | 7.56 | <.001 |
| PC aa C38:0 | 17 | 2.39 | 0.78 | 1.98 | 0.64 | -0.41 | 0.06 | 6.52 | <.001 |
| PC aa C38:3 | 17 | 48.06 | 9.61 | 27.06 | 7.57 | -21 | 2.1 | 9.99 | <.001 |
| PC aa C38:4 | 17 | 88.69 | 15.65 | 74.78 | 14.24 | -13.91 | 2.22 | 6.26 | <.001 |
| PC aa C38:5 | 17 | 50.8 | 10.03 | 39.72 | 9.67 | -11.08 | 2.07 | 5.35 | <.001 |
| PC aa C40:2 | 17 | 0.23 | 0.04 | 0.16 | 0.04 | -0.07 | 0.01 | 6.86 | <.001 |
| PC aa C40:3 | 17 | 0.41 | 0.08 | 0.3 | 0.06 | -0.11 | 0.02 | 5.65 | <.001 |
| PC aa C40:4 | 17 | 2.59 | 0.57 | 1.76 | 0.41 | -0.83 | 0.13 | 6.37 | <.001 |
| PC aa C40:5 | 17 | 7.44 | 1.51 | 5.22 | 1.62 | -2.22 | 0.37 | 5.97 | <.001 |
| PC aa C40:6 | 17 | 23.54 | 8.78 | 17.98 | 6.37 | -5.56 | 0.97 | 5.74 | <.001 |
| PC aa C42:1 | 17 | 0.2 | 0.06 | 0.19 | 0.06 | -0.01 | 0.01 | 1.85 | 0.05 |
| PC aa C42:2 | 17 | 0.17 | 0.04 | 0.12 | 0.03 | -0.05 | 0.01 | 7.18 | <.001 |
| PC aa C42:4 | 17 | 0.11 | 0.02 | 0.09 | 0.02 | -0.02 | 0 | 5.01 | <.001 |
| PC aa C42:5 | 17 | 0.25 | 0.09 | 0.17 | 0.05 | -0.08 | 0.02 | 5.16 | <.001 |
| PC aa C42:6 | 17 | 0.37 | 0.09 | 0.22 | 0.07 | -0.14 | 0.02 | 7.55 | <.001 |
| PC ae C30:0 | 17 | 0.4 | 0.18 | 0.21 | 0.06 | -0.19 | 0.04 | 5.09 | <.001 |
| PC ae C30:2 | 17 | 0.09 | 0.02 | 0.07 | 0.01 | -0.02 | 0 | 7.23 | <.001 |
| PC ae C32:1 | 17 | 3.53 | 0.73 | 3.19 | 0.67 | -0.34 | 0.09 | 3.88 | <.001 |
| PC ae C32:2 | 17 | 0.9 | 0.19 | 0.79 | 0.18 | -0.11 | 0.02 | 6.81 | <.001 |
| PC ae C34:0 | 17 | 1.94 | 0.64 | 1.15 | 0.33 | -0.79 | 0.12 | 6.33 | <.001 |
| PC ae C34:1 | 17 | 12.28 | 3.15 | 9.67 | 2.07 | -2.61 | 0.45 | 5.75 | <.001 |
| PC ae C34:2 | 17 | 14.5 | 3.17 | 9.59 | 2.39 | -4.92 | 0.75 | 6.52 | <.001 |
| PC ae C34:3 | 17 | 9.99 | 2.19 | 7.45 | 2.01 | -2.54 | 0.56 | 4.51 | <.001 |
| PC ae C36:0 | 17 | 0.89 | 0.24 | 0.75 | 0.2 | -0.14 | 0.03 | 4.44 | <.001 |
| PC ae C36:1 | 17 | 8.8 | 2.19 | 5.61 | 1.4 | -3.19 | 0.39 | 8.17 | <.001 |
| PC ae C36:2 | 17 | 16.04 | 3.24 | 10.38 | 2.55 | -5.66 | 0.61 | 9.31 | <.001 |
| PC ae C36:3 | 17 | 8.63 | 1.94 | 5.14 | 1.45 | -3.49 | 0.46 | 7.53 | <.001 |
| PC ae C36:4 | 17 | 17.2 | 5.49 | 12.94 | 3.19 | -4.26 | 0.89 | 4.76 | <.001 |
| PC ae C36:5 | 17 | 12.27 | 4.97 | 9.22 | 2.58 | -3.05 | 0.84 | 3.63 | <.001 |
| PC ae C38:0 | 17 | 2.17 | 0.75 | 1.24 | 0.45 | -0.93 | 0.13 | 7.13 | <.001 |
| PC ae C38:2 | 17 | 1.97 | 0.42 | 1.16 | 0.35 | -0.81 | 0.1 | 8.05 | <.001 |
| PC ae C38:3 | 17 | 4.05 | 1.02 | 2.43 | 0.66 | -1.61 | 0.17 | 9.32 | <.001 |
| PC ae C38:4 | 17 | 11.58 | 2.81 | 9.42 | 1.99 | -2.15 | 0.41 | 5.32 | <.001 |
| PC ae C38:5 | 17 | 15.39 | 3.86 | 13.67 | 2.88 | -1.72 | 0.54 | 3.18 | 0.01 |
| PC ae C38:6 | 17 | 7.19 | 2.15 | 5.43 | 1.73 | -1.76 | 0.24 | 7.22 | <.001 |
| PC ae C40:1 | 17 | 0.99 | 0.28 | 0.66 | 0.21 | -0.33 | 0.06 | 5.97 | <.001 |
| PC ae C40:2 | 17 | 1.74 | 0.38 | 1.44 | 0.42 | -0.3 | 0.05 | 5.9 | <.001 |
| PC ae C40:3 | 17 | 0.91 | 0.18 | 0.71 | 0.16 | -0.2 | 0.03 | 6.63 | <.001 |
| PC ae C40:4 | 17 | 1.79 | 0.38 | 1.44 | 0.28 | -0.35 | 0.06 | 5.88 | <.001 |
| PC ae C40:6 | 17 | 3.88 | 1.1 | 3.41 | 1.08 | -0.47 | 0.13 | 3.53 | <.001 |
| PC ae C42:1 | 17 | 0.24 | 0.05 | 0.18 | 0.04 | -0.05 | 0.01 | 7.44 | <.001 |
| PC ae C42:2 | 17 | 0.46 | 0.11 | 0.29 | 0.08 | -0.17 | 0.03 | 6.73 | <.001 |
| PC ae C42:3 | 17 | 0.64 | 0.13 | 0.48 | 0.12 | -0.16 | 0.02 | 7.11 | <.001 |
| PC ae C42:4 | 17 | 0.67 | 0.14 | 0.51 | 0.13 | -0.15 | 0.03 | 5.86 | <.001 |
| PC ae C42:5 | 17 | 1.58 | 0.35 | 1.73 | 0.37 | 0.15 | 0.04 | -3.57 | <.001 |
| PC ae C44:4 | 17 | 0.28 | 0.05 | 0.23 | 0.06 | -0.05 | 0.01 | 4.04 | <.001 |
| PC ae C44:5 | 17 | 1.24 | 0.24 | 1.39 | 0.29 | 0.15 | 0.04 | -4.04 | <.001 |
| SM C18:0 | 17 | 20.77 | 4.24 | 24.81 | 6.42 | 4.04 | 1.29 | -3.14 | 0.01 |
| SM C18:1 | 17 | 10.18 | 2.22 | 12.98 | 3.62 | 2.81 | 0.61 | -4.6 | 0.01 |
| SM C20:2 | 17 | 0.31 | 0.11 | 0.42 | 0.14 | 0.11 | 0.02 | -5.64 | 0.01 |
| SM C24:0 | 17 | 14.83 | 3.03 | 9.05 | 2.17 | -5.78 | 0.45 | 12.95 | 0.01 |
| SM (OH) C14:1 | 17 | 6.88 | 1.66 | 6.05 | 1.59 | -0.83 | 0.21 | 3.96 | 0.01 |
| SM (OH) C22:1 | 17 | 9.89 | 1.8 | 6.11 | 1.75 | -3.78 | 0.32 | 11.96 | 0.01 |
| SM (OH) C22:2 | 17 | 8.52 | 1.93 | 6.68 | 1.68 | -1.85 | 0.29 | 6.48 | 0.01 |
| SM (OH) C24:1 | 17 | 0.96 | 0.23 | 0.64 | 0.15 | -0.33 | 0.03 | 9.54 | 0.01 |
| H1 | 17 | 5241.29 | 724.27 | 4896.24 | 643.11 | -345.06 | 163.3 | 2.11 | 0.05 |

**Note.** *P-value based on the log-transformed analysis

**Table S7. Metabolites significantly associated with weight loss in the Intercept.**

| **Metabolite** | **Unadjusted** |  | **Adjusted** | |
| --- | --- | --- | --- | --- |
|  | **β(95%CI)** | ***p*** | **β(95%CI)** | ***p*** |
| C12 | -10.24(-20.5;0.03) | 0.051 | -11.26(-21.99;-0.52) | 0.042 |
| C14:1 | -9.1(-15.61;-2.58) | 0.009 | -10.85(-16.22;-5.49) | 0.001 |
| C14:2 | -10.88(-19.55;-2.22) | 0.017 | -11.56(-18.13;-5) | 0.003 |
| C16 | -5.62(-9.69;-1.55) | 0.010 | -6.07(-10.5;-1.63) | 0.012 |
| C18:1 | -5.19(-8.86;-1.52) | 0.009 | -5.78(-9.26;-2.3) | 0.004 |
| C18:2 | -7.3(-14.52;-0.08) | 0.048 | -8.6(-16.46;-0.74) | 0.035 |
| C2 | -5.8(-10.48;-1.12) | 0.018 | -7(-12.54;-1.46) | 0.018 |
| C3 | 4.12(0.57;7.68) | 0.026 | 5.55(1.85;9.26) | 0.007 |
| PC aa C34:3 | 10.78(3.6;17.95) | 0.006 | 12.79(5.09;20.49) | 0.004 |
| PC aa C34:4 | 11.55(2.69;20.42) | 0.014 | 13.28(2.89;23.67) | 0.017 |
| PC aa C36:1 | 3.75(-1.94;9.44) | 0.181 | 5.81(0.53;11.09) | 0.034 |
| PC aa C36:2 | 3.8(-0.93;8.54) | 0.108 | 5.98(1.34;10.63) | 0.016 |
| PC aa C36:3 | 3.59(-0.78;7.97) | 0.101 | 5.35(0.85;9.85) | 0.024 |
| PC ae C34:2 | 2.23(-2.42;6.88) | 0.323 | 4.58(0.53;8.63) | 0.030 |
| PC ae C36:0 | -2.81(-5.1;-0.52) | 0.019 | -2.68(-5.19;-0.17) | 0.039 |
| PC ae C38:2 | 2.74(-3.03;8.5) | 0.328 | 5.57(0.12;11.01) | 0.046 |
| PC ae C40:5 | -3.82(-6.21;-1.43) | 0.004 | -3.55(-5.94;-1.16) | 0.007 |
| SMC16:0 | -2.87(-4.34;-1.39) | 0.001 | -2.22(-3.59;-0.85) | 0.004 |
| SMC16:1 | -2.54(-4;-1.09) | 0.002 | -2.1(-3.74;-0.47) | 0.016 |
| SMC18:0 | -6.18(-9.5;-2.86) | 0.001 | -6.58(-10.35;-2.82) | 0.003 |
| SMC18:1 | -5.09(-8.04;-2.14) | 0.002 | -5.4(-8.91;-1.89) | 0.006 |
| SMC20:2 | -6.09(-10.65;-1.54) | 0.012 | -7.31(-12.77;-1.85) | 0.013 |
| SMOhC16:1 | -5.06(-7.76;-2.35) | 0.001 | -4.96(-7.88;-2.04) | 0.003 |

**Note.** Analysis using residuals from Log transformed metabolites with fixed effect for sex. The multivariable model included additional adjustment for age, BMI, residuals for WC (removing BMI effect) and residuals for WHR (removing BMI and WC effect).

**Figure S1. Overall *R*_partial_2 and weighted *R*_partial_2 for each covariate and BMI in the discovery set.** Note. Similar results were observed for the EPIC replication, colorectal, and endometrial sets.

**Figure S2. Overall *R*_partial_2 and weighted *R*_partial_2 for each covariate and WC in the discovery set.** Note. Similar results were observed for the EPIC replication, colorectal, and endometrial sets.

**Figure S3. Overall *R*_partial_2 and weighted *R*_partial_2 for each covariate and WHR in the discovery set. Note.** Similar results were observed for the EPIC replication, colorectal, and endometrial sets.

**Figure S4. Overall *R*_partial_2 and weighted *R*_partial_2 for each covariate and weight loss in the Intercept.**

**
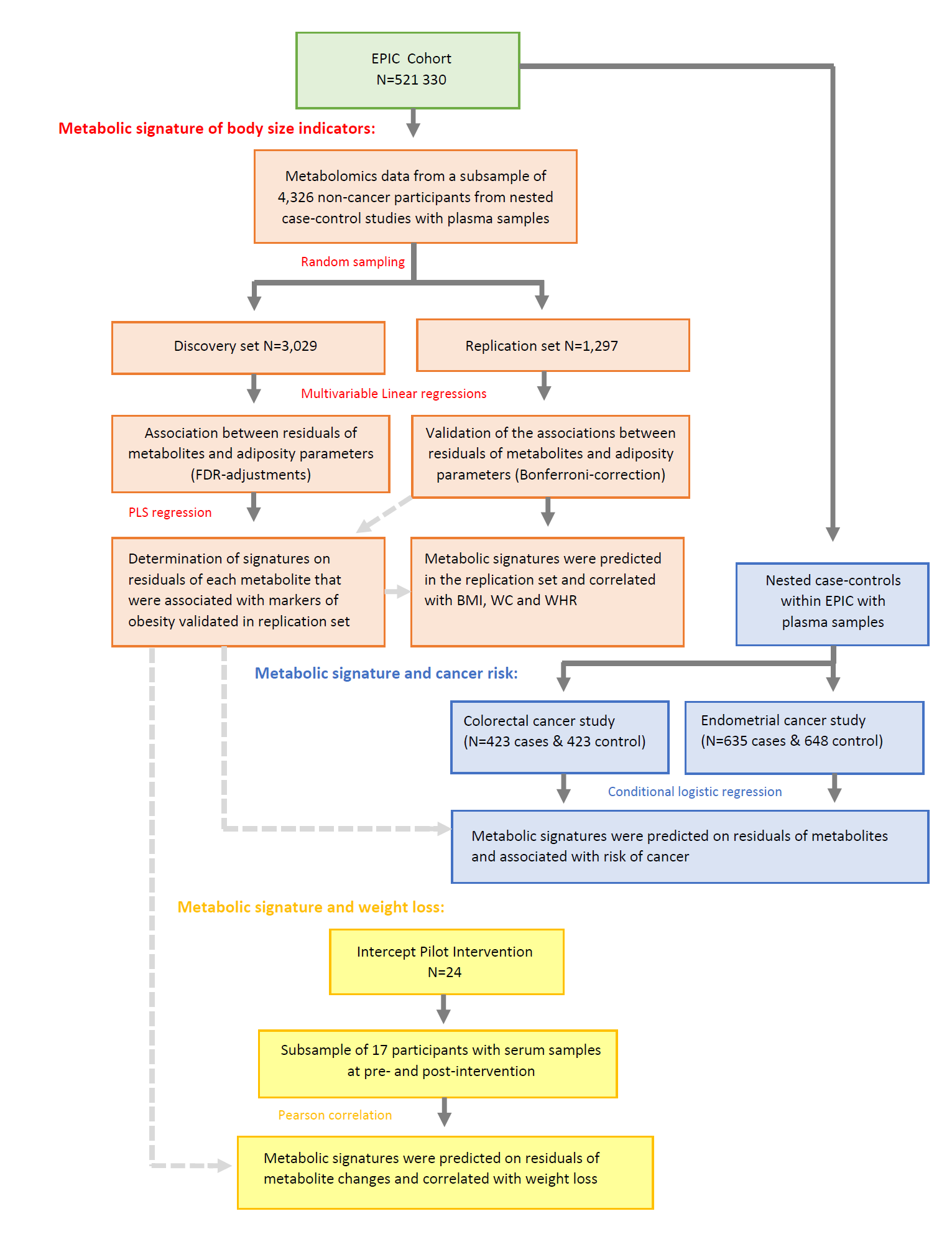
**

**Figure S5. Flow-diagram with the main methodological steps of the study**

1.
2.
3.

**Figure S6. Overall *R*_partial_2 and weighted *R*_partial_2 for residuals of metabolites that had their association with markers of obesity validated and anthropometric variables in the discovery set. (a) Metabolites associated to BMI. (b) Metabolites associated to WC. (c) Metabolites associated to WHR.**


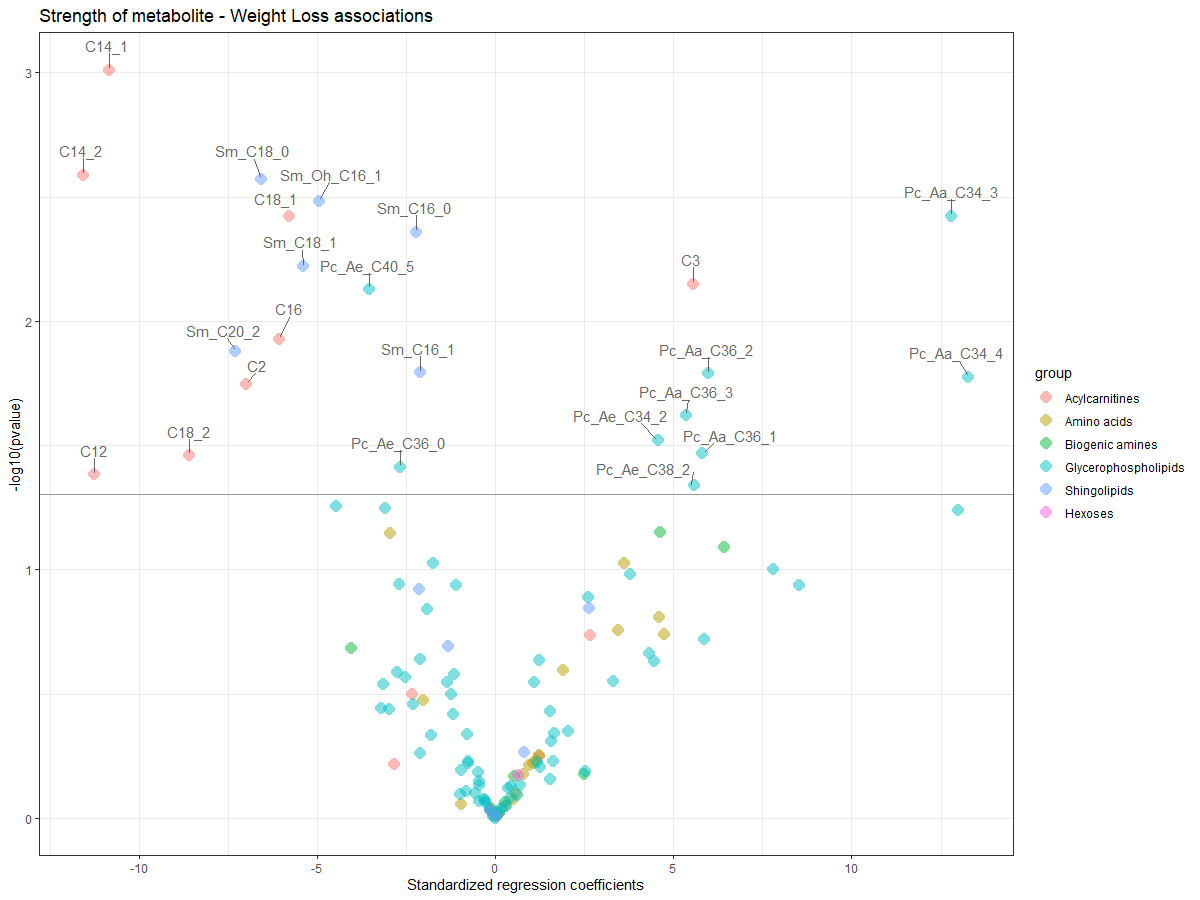


**Figure S7. Smile plot of the associations between metabolites with weight loss in the Intercept.** Note. Analysis using residuals from Log transformed metabolites with fixed effect for sex. Models were adjusted for age, BMI, residuals for WC (removing BMI effect) and residuals for WHR (removing BMI and WC effect). The metabolites above the horizontal line showed a significant association with the anthropometric measure (p<0.05).


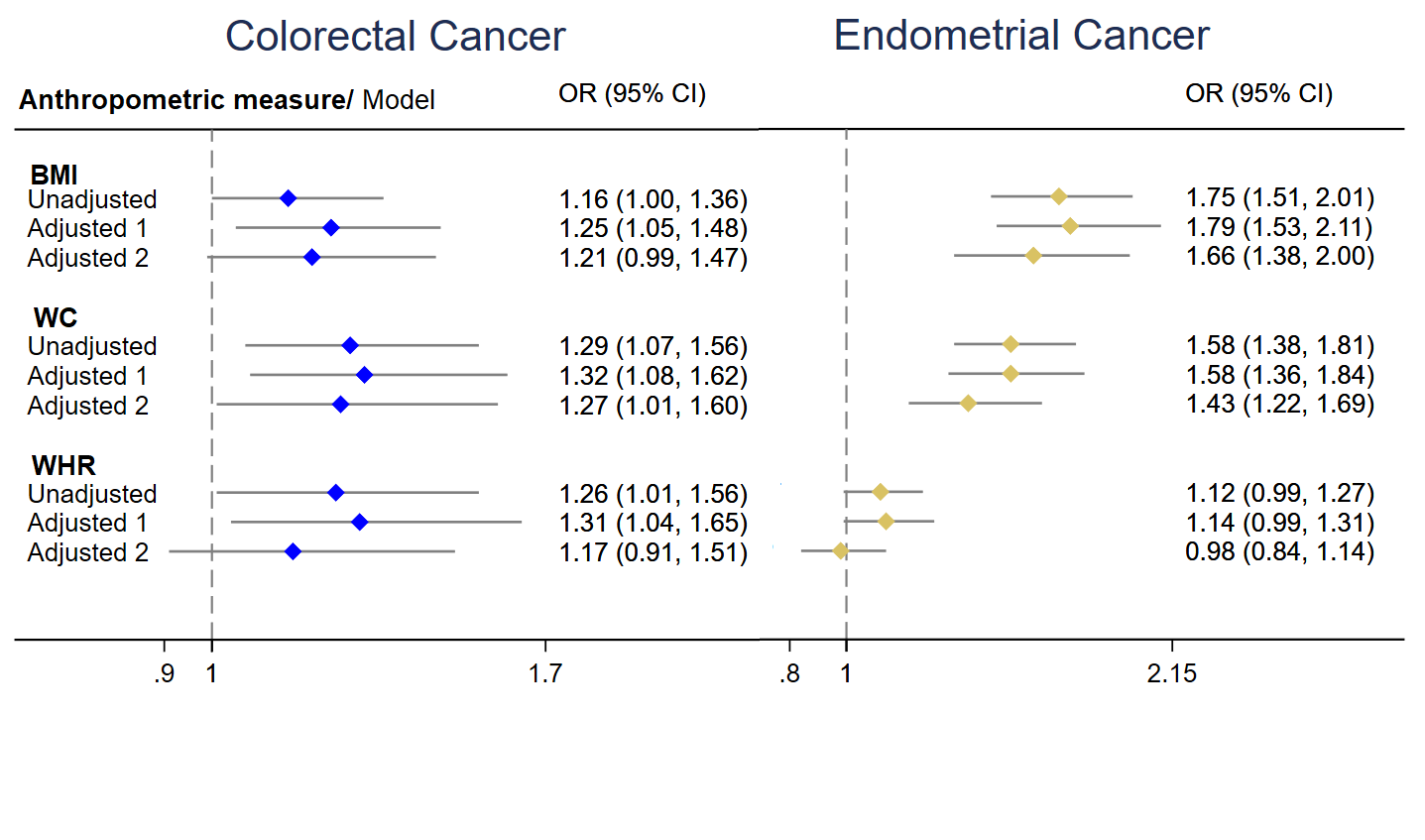


**Figure S8 Association of colorectal and endometrial cancers with the anthropometric measures of obesity.** Note. Model 1 was adjusted for height, physical activity, smoking status, education level, consumption of alcohol, total energy, red and processed meats, fish and shellfish, age at blood collection, and fasting status. For endometrial cancer model 1 was further adjusted to menopause status, hormonal therapy, oral contraceptive use, age at first menstrual period and age at first full pregnancy, while for colorectal cancer model 1 was further adjusted for fibre and calcium intake. Models 2 included the adjustments from model 1 plus the metabolic signature of the anthropometric measure.
